# Global burden of chronic kidney disease and its attributable risk factors (1990-2021): an analysis based on the global burden of disease study

**DOI:** 10.3389/fendo.2025.1563246

**Published:** 2025-07-03

**Authors:** Min-Jia Li, Hong-Yong Liu, Yun-Qiang Zhang, Sheng-Rong Li, Jian-Hong Zhang, Rong Li

**Affiliations:** Department of Nephrology, The Third Affiliated Hospital of Sun Yat-Sen University, Yuedong Hospital, Meizhou, Guangdong, China

**Keywords:** chronic kidney disease, global burden of disease, prevalence, mortality, disability-adjusted life years, risk factors

## Abstract

**Background:**

Chronic kidney disease is a global health challenge, especially in resource-limited regions. Understanding its burden and key risk factors is crucial for effective interventions.

**Methods:**

Data from the Global Burden of Disease Study (1990–2021) covering 204 countries were analyzed to assess trends in prevalence, mortality, and disability-adjusted life years for chronic kidney disease, alongside geographic, age, sex, and risk factor patterns.

**Findings:**

In 2021, an estimated 674 million individuals were affected globally. Although the global age-standardized prevalence rate declined slightly by 0.8% since 1990, substantial disparities persisted. High-income North America’s prevalence remained stable yet showed high mortality (20.6 per 100,000) and increased DALYs (508.8 per 100,000). In contrast, East Asia’s prevalence fell by 11.7%, accompanied by notable reductions in mortality (11.1 per 100,000) and DALYs (322.4 per 100,000). Regions with low socio-demographic indices, including parts of sub-Saharan Africa and South Asia, bore the greatest burdens, with persistently high prevalence (exceeding 9000 per 100,000) and rising DALYs. Some countries, such as Guatemala, experienced rapid prevalence increases, while others, like Italy, achieved substantial reductions. Similarly, mortality trends varied: Ukraine saw steep increases, while Poland showed marked improvements. DALY burdens also diverged, with El Salvador experiencing large increases, and Kuwait recording significant declines. Prevalence peaked at ages 65–69 in males and 70–74 in females, with female rates surpassing male rates from the early thirties onward. High fasting plasma glucose contributed 36% of DALYs globally, heavily affecting the Caribbean and high-income North America. High systolic blood pressure and high body mass index were particularly influential in Central Europe and high-income North America, respectively. Low fruit intake strongly affected the Middle East and East Asia.

**Interpretation:**

These findings underscore persistent global inequalities in CKD outcomes, necessitating region-specific strategies and strengthened healthcare capacity to mitigate the burden.

## 1 Introduction

Chronic kidney disease (CKD) is a major global public health concern (1). Estimates from the Global Burden of Disease (GBD) study show that approximately 697 million people worldwide are affected by CKD (2), highlighting its significant health and economic impacts. This issue is especially severe in low- and middle-income countries (LMICs) (3), largely due to aging populations, rising obesity rates, and the increased prevalence of chronic conditions such as diabetes and hypertension (4–6). Moreover, CKD substantially increases the risk of cardiovascular disease (7), further complicating health outcomes for those affected.

Socioeconomic disparities in CKD prevalence are pronounced. In LMICs, scarce healthcare resources result in lower rates of early screening and diagnosis (8, 9), with many patients identified only at advanced disease stages. This delay exacerbates healthcare system burdens and amplifies the impact of established risk factors like obesity, diabetes, and hypertension (10–12). Additionally, environmental pollutants, including PM2.5 particulates and heavy metals, are now recognized as significant CKD risk contributors (13, 14). Comprehensive understanding of CKD’s global epidemiological patterns and its key risk factors is thus critical for guiding effective public health policies and interventions.

This study aims to systematically evaluate the global trends and primary risk factors of CKD, with a special focus on the influence of socioeconomic context, environmental factors, and lifestyle on CKD prevalence and mortality. By analyzing data from the GBD database from 1990 to 2021 (15), we seek to identify high-risk populations and regions, providing policymakers with the evidence needed to design targeted interventions to reduce the global CKD burden and improve patient outcomes.

## 2 Methods

### 2.1 Study design and population

#### 2.1.1 Data sources and sample selection

This study utilized data from the Global Burden of Disease (GBD) database, encompassing information on chronic kidney disease (CKD) from 1990 to 2021 across 204 countries and 21 regions. The dataset included measures of prevalence (Prevalent Cases), mortality (Deaths), and disability-adjusted life years (DALYs) (16). All data underwent standardized processing at the source, ensuring accuracy, comparability, and consistency across geographic and temporal scales.

#### 2.1.2 Data processing

Data processing involved the independent standardization and reformatting of prevalence, mortality, and DALY metrics. We utilized R software (version 4.3.3) along with its associated packages, such as tidyverse and dplyr, for data cleaning, normalization, and unit conversion, standardizing all metrics to per 100,000 population. Key statistical indicators were recalculated to more accurately reflect recent regional trends and enable meaningful comparisons.

#### 2.1.3 Descriptive statistical analysis

Descriptive statistics were applied to the prevalence, mortality, and DALY data. We employed medians and 95% confidence intervals (CIs) to summarize central tendencies and quantify uncertainty. Additionally, stratified analyses by country and region were performed, involving the calculation of 95% CIs to highlight global and regional variations in CKD burden. These standardized and recalculated indicators provided a comprehensive overview of CKD-related health burdens across varied geographies.

#### 2.1.4 Geographic information system and visualization

To depict the spatial distribution and temporal trends of CKD, we utilized geographic information system (GIS) techniques, leveraging R packages like ggplot2 and sf for visualization. By mapping the prevalence and mortality rates for 1990 and 2021, we identified high-risk regions and visualized changes over time. This analysis aids in informing resource allocation and shaping policy development.

#### 2.1.5 Socio-demographic index correlation analysis

We conducted Pearson correlation analyses to explore the relationship between the Socio-Demographic Index (SDI) and CKD-related disability-adjusted life years (DALYs). This analysis assessed data across 204 countries, offering insights into the interaction between socioeconomic factors and the burden of CKD. It helped to elucidate the complex impacts of demographic and economic conditions on disease outcomes.

#### 2.1.6 Risk factor analysis

We extracted data on 84 health risk factors from the GBD database and visualized their relative contributions to CKD burden in different regions using bar charts. This comparative analysis enabled the identification of key risk factors, which aids in guiding targeted public health interventions and tailoring preventive measures to the specific contexts of various regions.

#### 2.1.7 Data analysis tools and statistical software

All analyses were conducted using R version 4.3.3 along with associated packages, such as tidyverse for data processing and integration, ggplot2 and sf for GIS-based visualization, and the stats package for statistical analyses. GIS tools (via the sf package) were used to visualize regional disparities in disease burden, enhancing understanding of geographic patterns in CKD prevalence and DALYs. We adhered strictly to GBD data usage guidelines and upheld best practices for reproducible research. If necessary, all code and analytical procedures can be made available in a suitable repository to support the verification and replication of our findings.

#### 2.1.8 Ethical approval

This study utilized publicly available, anonymized data from the Global Burden of Disease database. No human subjects were directly involved, and no ethical approval or informed consent was required.

## 3 Results

Note: All prevalence, mortality, and DALYs data in this section are presented as “per 100,000 population”.

### 3.1 Global and regional burden of chronic kidney disease

**Table 1** summarizes the global and regional burden of CKD from 1990 to 2021. In 2021, 674 million individuals were affected worldwide, with an age-standardized prevalence rate (ASPR) of 8006, marking a 0.8% decrease from 1990. Despite this, significant regional disparities persist.

**Table 1:** Prevalence, Mortality, and DALYs by Region and Globally.

Location	Prevalence (95% UI)			Deaths (95% UI)			DalYs (95% UI)		
	No in millions (95% UI)	ASRs per 100000 (95% UI)	Percentage change in ASRs from 1990 to 2021	No in millions (95% UI)	ASRs per 100000 (95% UI)	Percentage change in ASRs from 1990 to 2021	No in millions (95% UI)	ASRs per 100000 (95% UI)	Percentage change in ASRs from 1990 to 2021
Global	673.7 (629.1,722.4)	8006 (7482.1,8575.6)	-0.8 (-1.9,0.1)	1527.6 (1389.4,1638.9)	18.5 (16.7,19.9)	24.5 (10.2,33.5)	44453.7 (40840.8,48508.5)	529.6 (486.2,577.4)	10.4 (0.8,17.8)
High-income Asia Pacific	29.3 (27.3,31)	7920.7 (7393.7,8457.4)	-7.5 (-8.5,-6.6)	63.5 (50.4,71)	9.7 (8.1,10.7)	-22.8 (-28.7,-18.6)	1146.6 (990.3,1268.3)	235.6 (206,259.6)	-25.4 (-29.1,-22.3)
High-income North America	42.5 (39.4,45.2)	7434.7 (6959.3,7911.4)	0.5 (-0.6,1.8)	143.7 (124.8,154.8)	20.6 (18.1,22)	147 (139.1,155.4)	3121 (2864.2,3351.6)	508.8 (467,545.4)	90.8 (82.4,100)
Western Europe	41.6 (39.1,43.9)	5226.2 (4924.4,5544.2)	-4.5 (-6.2,-3)	133.5 (109.6,148.2)	10.7 (8.9,11.8)	29 (18.4,37.6)	2361.3 (2067.1,2638.2)	241.7 (209.8,271.1)	3.2 (-1.7,8.5)
Australasia	2.8 (2.6,3)	5910 (5548.2,6301.6)	-2.9 (-5.7,0.1)	6 (5,6.5)	9.6 (8.2,10.5)	13.7 (6.1,20.4)	113.7 (101.5,125.3)	216.6 (193.2,239.2)	2.6 (-2.5,7.1)
Andean Latin America	3.7 (3.5,4)	5946.1 (5524.8,6371.5)	3.1 (0.5,5.2)	21.6 (17.9,25.8)	37.7 (31.3,44.9)	32 (7.7,63.3)	524.2 (434.4,625)	872.4 (723.5,1037.9)	15.7 (-4.2,41.2)
Tropical Latin America	19.3 (18,20.7)	7576.2 (7081,8107.5)	-2.4 (-3.4,-1.4)	46.9 (42.5,49.4)	18.8 (17,19.9)	5 (-0.5,9.1)	1311.9 (1220.7,1401.2)	517 (480.6,552.1)	-8.1 (-12,-4.7)
Central Latin America	22.1 (20.7,23.4)	8642.9 (8089.3,9163.7)	2 (0.1,3.9)	104.4 (94.2,116.5)	42.4 (38.3,47)	51.6 (37.5,66.7)	2993.8 (2691.7,3371.7)	1171.1 (1054.8,1316.3)	52.5 (37.6,68.7)
Southern Latin America	4.9 (4.5,5.2)	5970.4 (5543.1,6415)	3.2 (0.7,6.1)	21.6 (19.5,23)	23.8 (21.6,25.4)	-8.3 (-14.1,-3)	441.1 (411.3,467.9)	515.2 (481.8,546.8)	-15.9 (-20.3,-11.5)
Caribbean	3.5 (3.2,3.7)	6633 (6181.3,7112.4)	2.4 (0.7,4)	13.9 (12.1,16.2)	25.8 (22.4,30.1)	37.6 (18.9,57.5)	385.3 (331.6,454.1)	735.8 (631,867.9)	30.2 (12.9,49.6)
Central Europe	11.5 (10.8,12.1)	6205.2 (5841,6596.2)	-2.6 (-4.1,-1)	21.6 (19.4,24)	9.4 (8.4,10.5)	-10.4 (-18.3,-1.7)	540 (472.2,612.7)	266.9 (233.4,306.2)	-22.1 (-28,-14.7)
Eastern Europe	27.3 (25.4,29.3)	9266.3 (8619.4,9989.5)	-0.7 (-1.5,0.2)	17.8 (16.1,19.9)	5.2 (4.7,5.8)	45.9 (33.3,59.9)	642.5 (562.9,727)	204.7 (180.4,232.1)	-1.3 (-7.9,7.1)
Central Asia	9.4 (8.7,10)	10698.2 (10022.9,11348.1)	0.5 (-1.3,2.1)	9.4 (8.3,10.5)	12.1 (10.7,13.5)	141.9 (101.3,186.6)	427.6 (377.5,490.7)	493.2 (436,565.5)	51.3 (37.2,68)
North Africa and Middle East	49.7 (45.8,53.6)	9180 (8523.5,9830.1)	0.7 (-0.3,1.8)	144.7 (126.2,162.7)	37.7 (32.7,42.4)	20.8 (-23.7,50.5)	3926 (3427.2,4413.6)	846.6 (747.2,948)	11.4 (-23.9,35.5)
South Asia	158.8 (147.2,171.6)	9565.3 (8903.7,10265.7)	-3.7 (-4.7,-2.8)	226 (192.5,264.2)	16.5 (14,19.3)	17.7 (-7.6,41.4)	8443.3 (7372.3,9681.8)	540.6 (473.8,620.4)	6 (-10.1,23.7)
Southeast Asia	73.6 (67.8,79.7)	10474.6 (9718.9,11301.7)	2.4 (1.6,3.2)	170 (148.9,190.5)	28.5 (24.8,31.9)	24.6 (2.4,43.3)	5703.3 (5028.7,6329)	846.3 (753.1,940.5)	12.5 (-2.1,26.6)
East Asia	122.8 (113.6,132.1)	6258.1 (5823.1,6729.8)	-11.7 (-13.5,-10.2)	217.3 (178,259.1)	11.1 (9.2,13.2)	-22.5 (-39.2,-5.5)	6486.2 (5538.1,7597.4)	322.4 (275.4,377.3)	-30.1 (-41.9,-18.5)
Oceania	0.8 (0.7,0.8)	7950.7 (7408.8,8567.7)	2.7 (0.7,4.4)	1.5 (1.3,1.9)	21.6 (18,26.4)	25.2 (-11.7,78.6)	65.8 (55.6,77.8)	699 (597.9,821.3)	19.8 (-9.5,60.4)
Western Sub-Saharan Africa	22.5 (20.9,24.2)	8324.3 (7782.3,8871)	0.2 (-0.9,1.2)	64.8 (53.5,76.1)	36.4 (31.1,42.8)	8.2 (-9.3,24.9)	2437.3 (1992.7,2893.6)	930.7 (788.4,1081.1)	0.2 (-13.2,13.9)
Eastern Sub-Saharan Africa	15 (13.7,16.4)	5821.3 (5404.5,6293.6)	2.5 (1.7,3.3)	60.9 (53.8,69.9)	40.1 (35.6,46)	-5.5 (-18.6,8.4)	2047.6 (1792,2374.2)	948.4 (838.7,1090.4)	-13.4 (-23,-0.9)
Central Sub-Saharan Africa	6.8 (6.4,7.3)	9165.2 (8640.8,9709.7)	-0.6 (-2.5,1.6)	21 (16.1,27.5)	43.7 (33.3,56.3)	2.4 (-22.2,33.4)	783.7 (622.6,996.5)	1124.7 (899,1436.1)	-2.9 (-23.8,24.9)
Southern Sub-Saharan Africa	6 (5.5,6.4)	9037.9 (8440.6,9647.1)	1 (-0.2,2.4)	17.4 (15.6,19.5)	34.4 (31.1,38.3)	65 (26.9,89.5)	551.5 (494.2,622.1)	896 (807.3,997)	43.6 (19.2,61.7)

In high-income North America, the ASPR stood at 7434.7, a modest increase of +0.5%, indicating a stable burden. However, the mortality rate was high at 20.6, and DALYs increased significantly by +90.8%. Conversely, East Asia saw a substantial reduction in its ASPR to 6258.1 (-11.7%), along with notable decreases in mortality (11.1) and DALYs (322.4), reflecting effective disease management. Sub-Saharan Africa continued to exhibit high ASPRs—8324.3 in the west, 9165.2 in the central, and 9037.9 in the south—with corresponding increases in DALYs. Central Asia (10698.2) and Southeast Asia (10474.6) reported the highest ASPRs, with Southeast Asia also showing particularly high mortality and DALYs.

In comparison, the high-income Asia Pacific region demonstrated a significant decrease in ASPR to 7920.7 (-7.5%), with substantial reductions in mortality (9.7) and DALYs (235.6), showcasing the benefits of a robust healthcare system. Overall, regions with low Socio-Demographic Indexes (SDIs) such as sub-Saharan Africa and South Asia experienced the heaviest CKD burdens, whereas high-income areas like the high-income Asia Pacific and Western Europe had relatively lighter burdens.

### 3.2 Trends in prevalence, mortality, and DALYs by region

**Supplementary Tables S1**-**S3** provide the trends in CKD prevalence, mortality, and DALYs worldwide and by region from 1990 to 2021, highlighting countries with the most significant increases or improvements.

#### 3.2.1 Prevalence trends

Between 1990 and 2021, countries with the most rapid increases in CKD prevalence included Guatemala (ASRs from 8065.5 to 8857.7, + 9.8%), Cameroon (8270.7 to 9043.6, + 9.3%), Tanzania (5368.9 to 5861.5, + 9.2%), Nicaragua (9675.2 to 10511.2, + 8.6%), and Oman (8427.7 to 9024.5, + 7.1%). These increases indicate a growing CKD burden across these nations.

**Figure 1:**
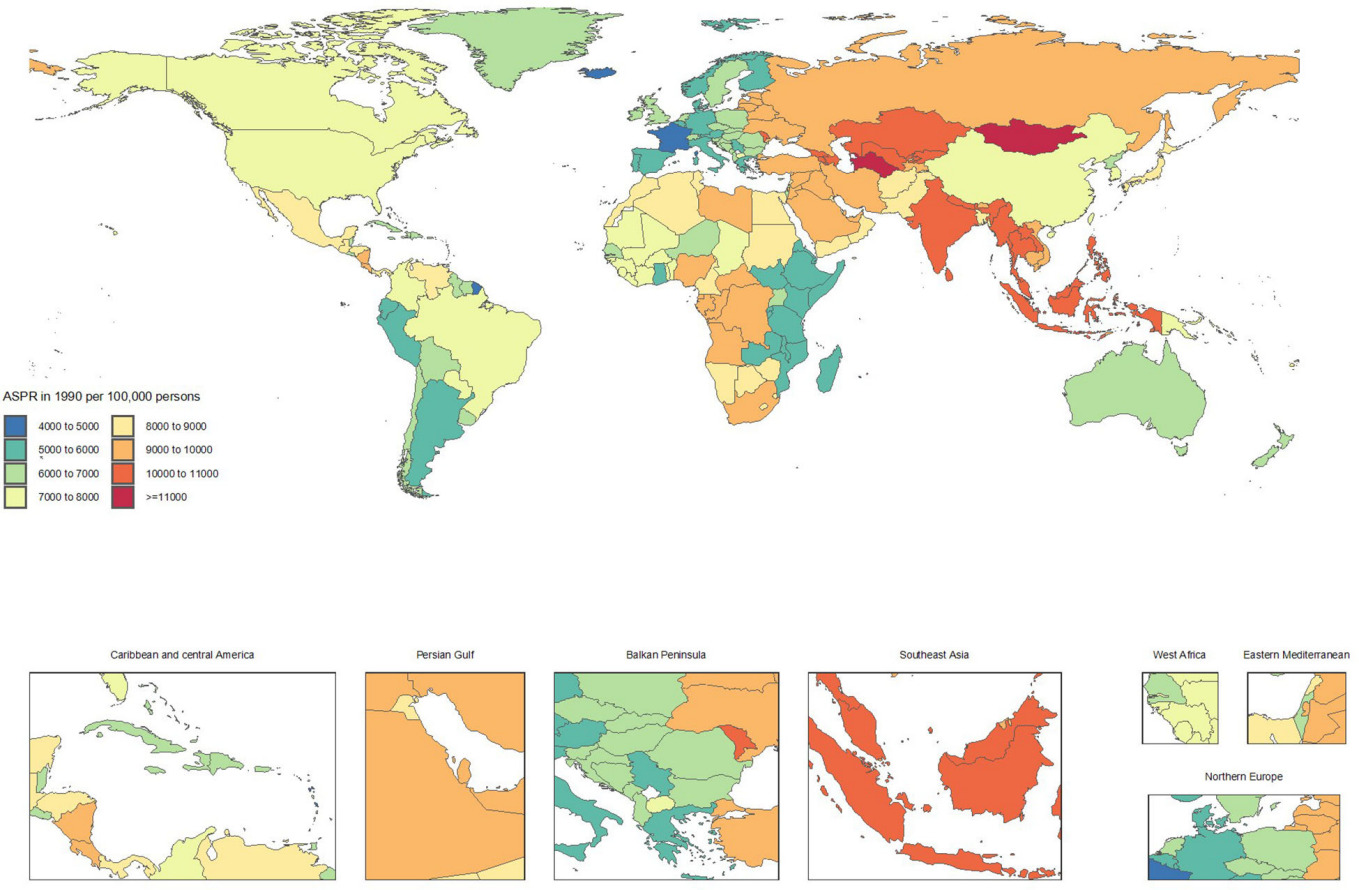
Age-standardized point prevalence of chronic kidney disease per 100,000 population in 1990, by country.

**Figure 2:**
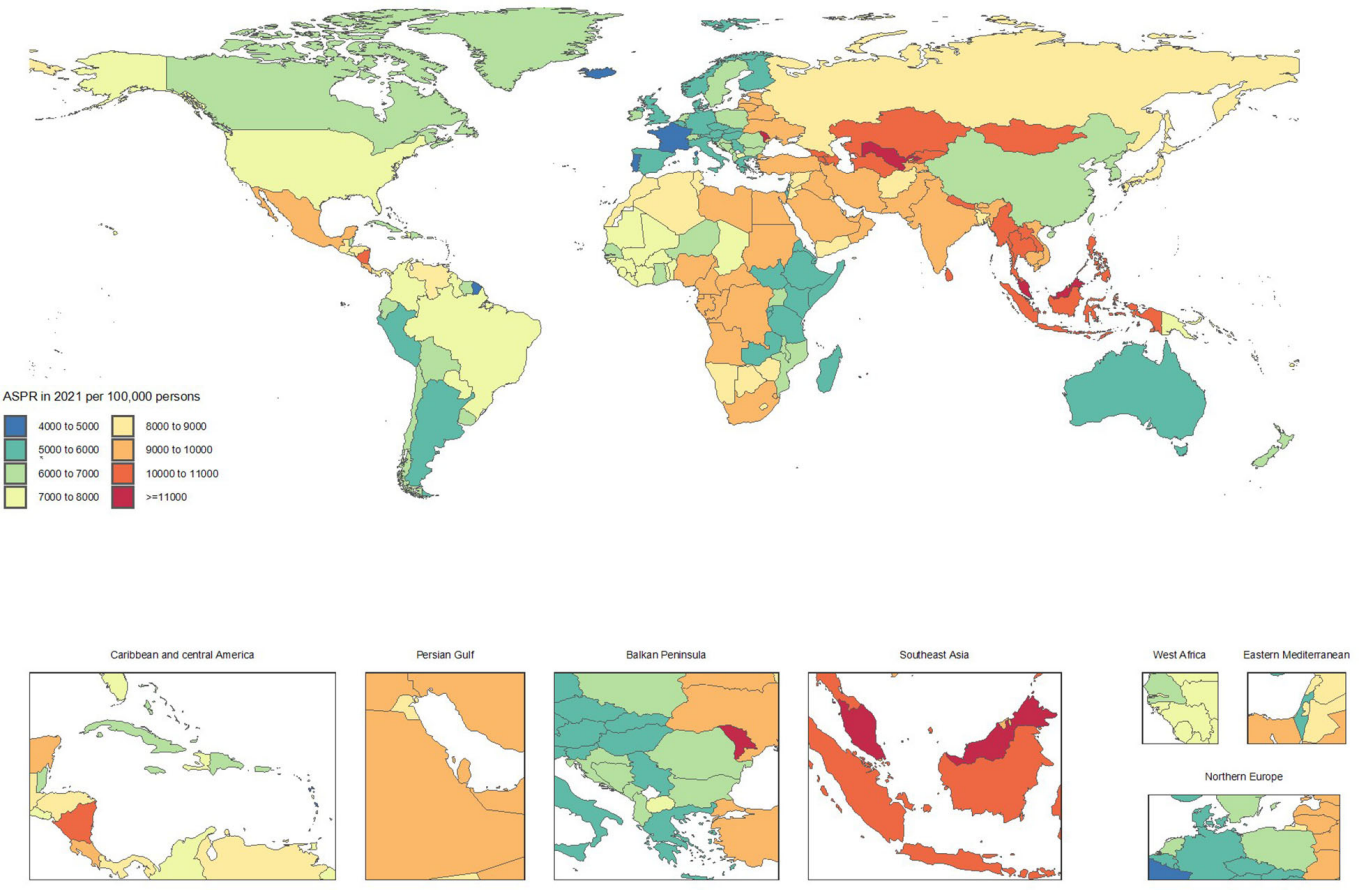
Age-standardized point prevalence of chronic kidney disease per 100,000 population in 2021, by country.

Conversely, countries showing significant improvement included Italy (ASRs from 5591.9 to 5061.3, -9.5%), Taiwan (Province of China) (7155.4 to 6571.2, -8.2%), Spain (5464.9 to 5022.8, -8.1%), Canada (7026.4 to 6524.4, -7.1%), and Portugal (5124.5 to 4777, -6.8%). The declining ASRs in these countries reflect substantial progress in CKD prevention and control (**Supplementary Table S1** and **Figures 1**, **2**).

#### 3.2.2 Mortality trends

From 1990 to 2021, countries with the fastest increases in mortality included Ukraine (ASMRs from 0.1 to 2.4, + 1714.7%), Armenia (1 to 10.3, + 953.1%), Georgia (3.3 to 12.3, + 272.5%), Belarus (0.8 to 2.4, + 182.4%), and Uzbekistan (5 to 14.2, + 182.1%). These substantial rises in mortality rates underscore a worsening CKD burden.

**Figure 3:**
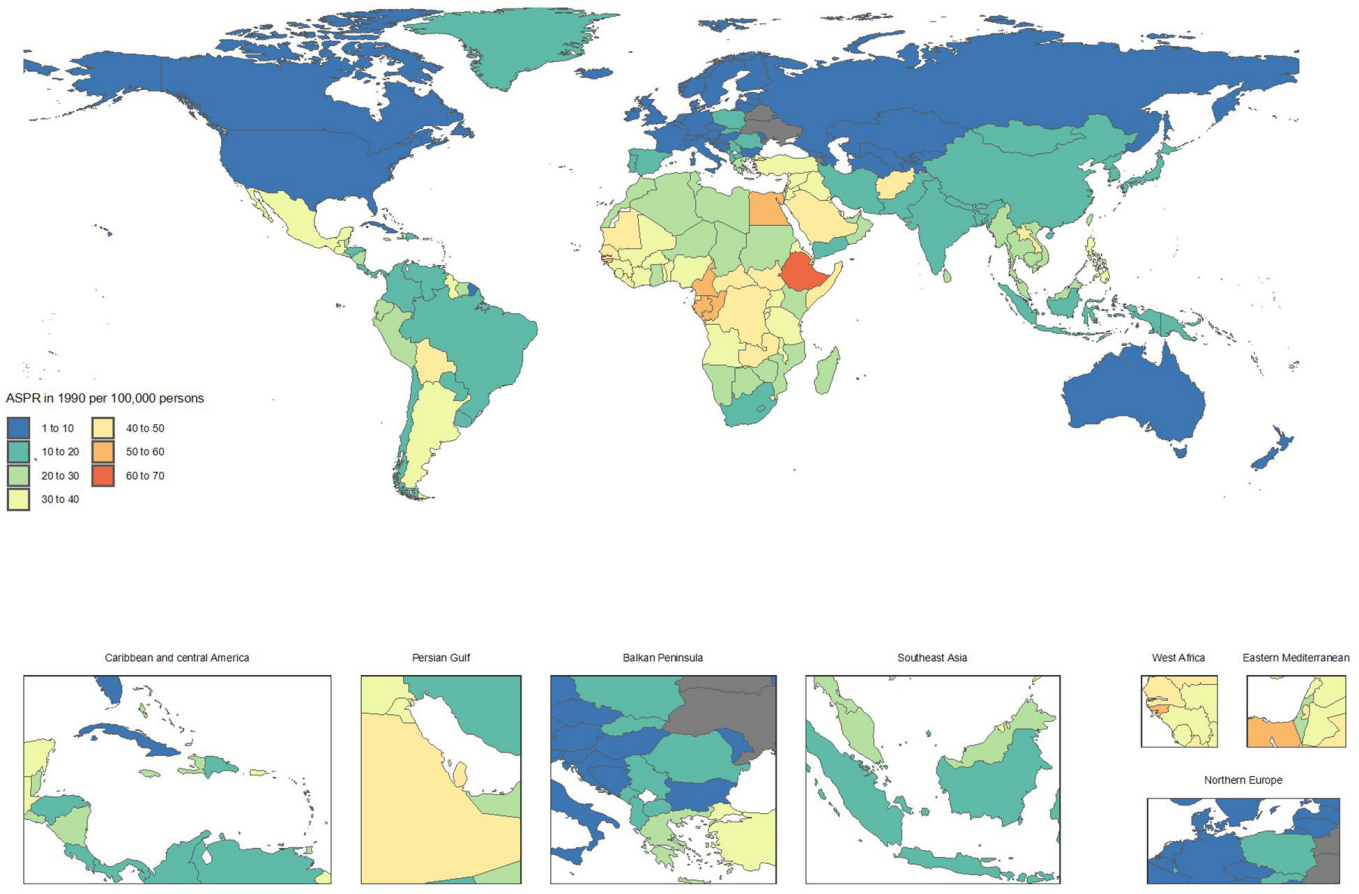
Age-standardized point deaths of chronic kidney disease per 100,000 population in 1990, by country.

**Figure 4:**
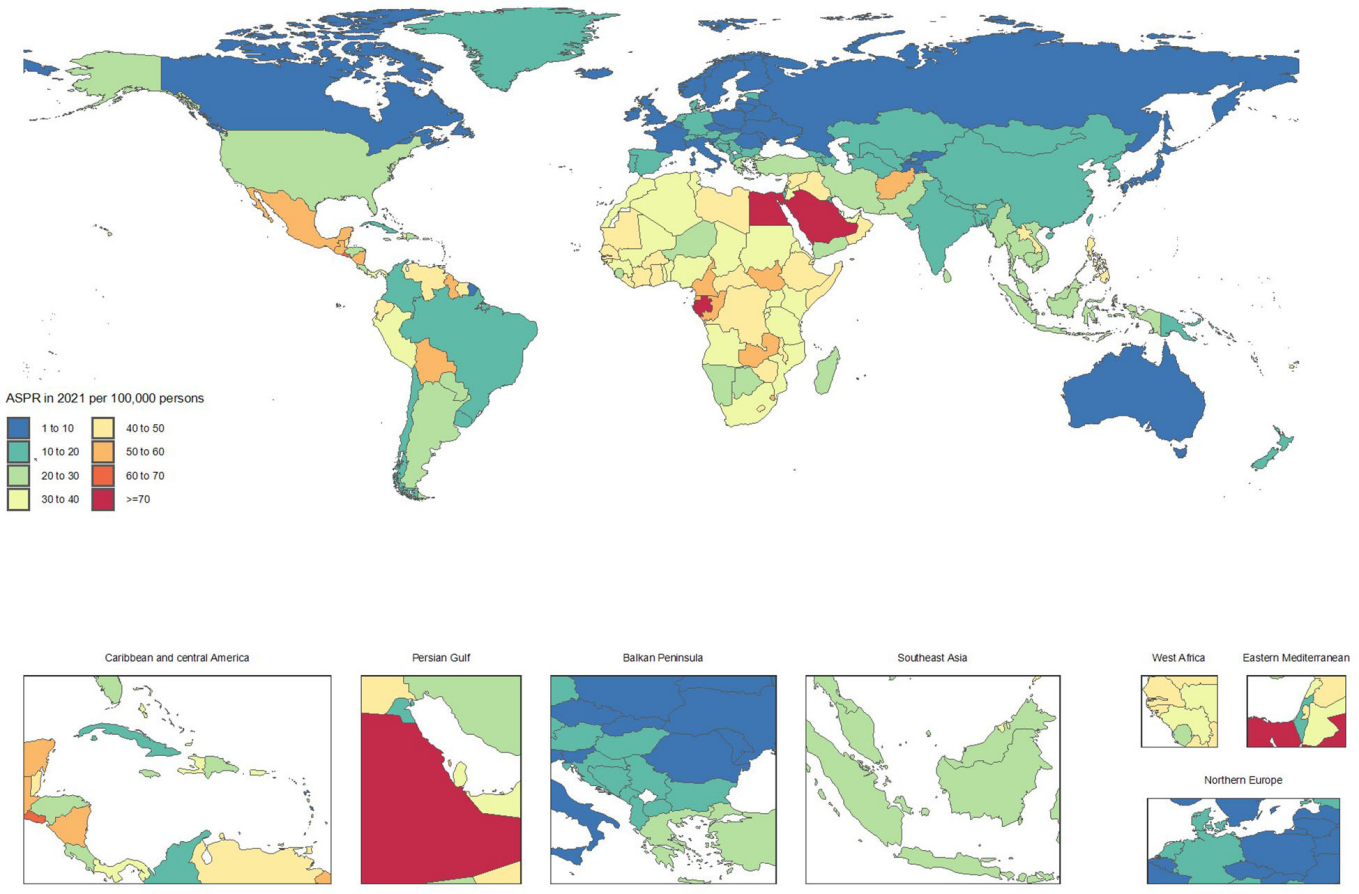
Age-standardized point deaths of chronic kidney disease per 100,000 population in 2021, by country.

In contrast, notable improvements in mortality were observed in Poland (ASMRs from 12.8 to 6.6, -48.5%), Kuwait (30.7 to 16, -47.9%), Cyprus (41.3 to 21.5, -47.8%), Republic of Korea (18.6 to 10.8, -41.8%), and the Maldives (48.4 to 28.9, -40.3%). These reductions highlight significant advancements in CKD management and treatment (**Supplementary Table S2** and **Figures 3**, **4**).

#### 3.2.3 DALYs trends

Between 1990 and 2021, countries experiencing the greatest increases in DALYs included El Salvador (ASRs from 784.3 to 1904, + 142.8%), Armenia (168.2 to 380.2, + 126%), Lesotho (560.6 to 1221.5, + 117.9%), the Venezuela(Bolivarian Republic of) (527.6 to 1087, + 106%), and American Samoa (953.2 to 1950.9, + 104.7%). These trends demonstrate a substantial worsening of the CKD burden.

Countries showing significant DALYs improvements included Kuwait (ASRs from 716.8 to 360.7, -49.7%), Poland (405.7 to 206.2, -49.2%), the Maldives (1347.6 to 719.8, -46.6%), Ethiopia (1765.7 to 966, -45.3%), and Cyprus (660.4 to 377.8, -42.8%). These reductions suggest remarkable success in comprehensive CKD prevention and intervention efforts.

### 3.3 Age and sex distribution

**Figure 5:**
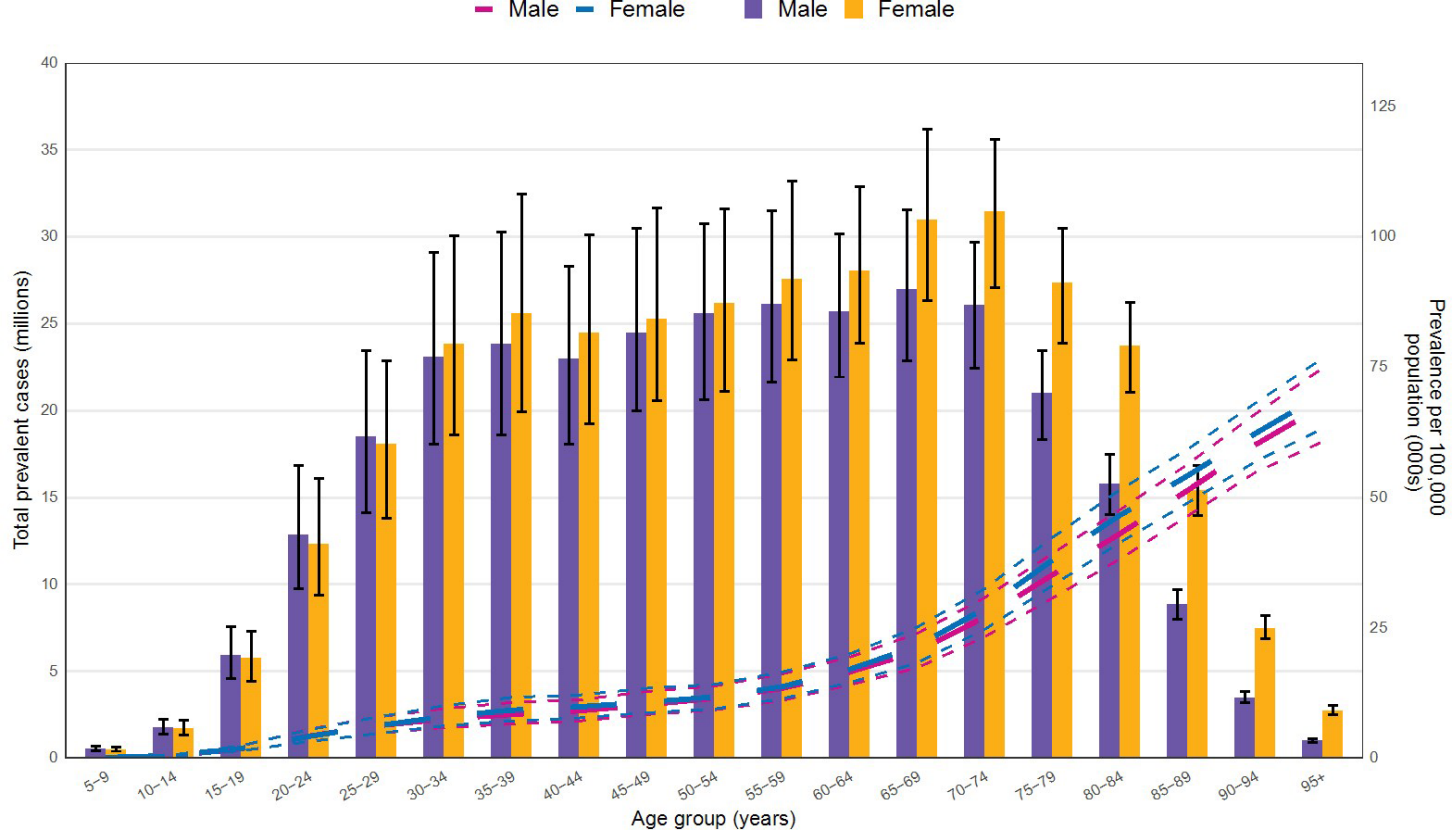
Age and Sex-Specific CKD Prevalence in 2021.

**Figure 5** illustrates the CKD prevalence by age and sex in 2021. Prevalence begins to increase noticeably among both sexes at ages 20-24. As age advances, the prevalence peaks at 65–69 years for males and at 70–74 years for females, subsequently declining for both sexes. Notably, starting from the 30–34 age group, the prevalence among females surpasses that of males, maintaining a consistently higher level thereafter.

### 3.4 SDI and DALYs correlation

**Figure 6:**
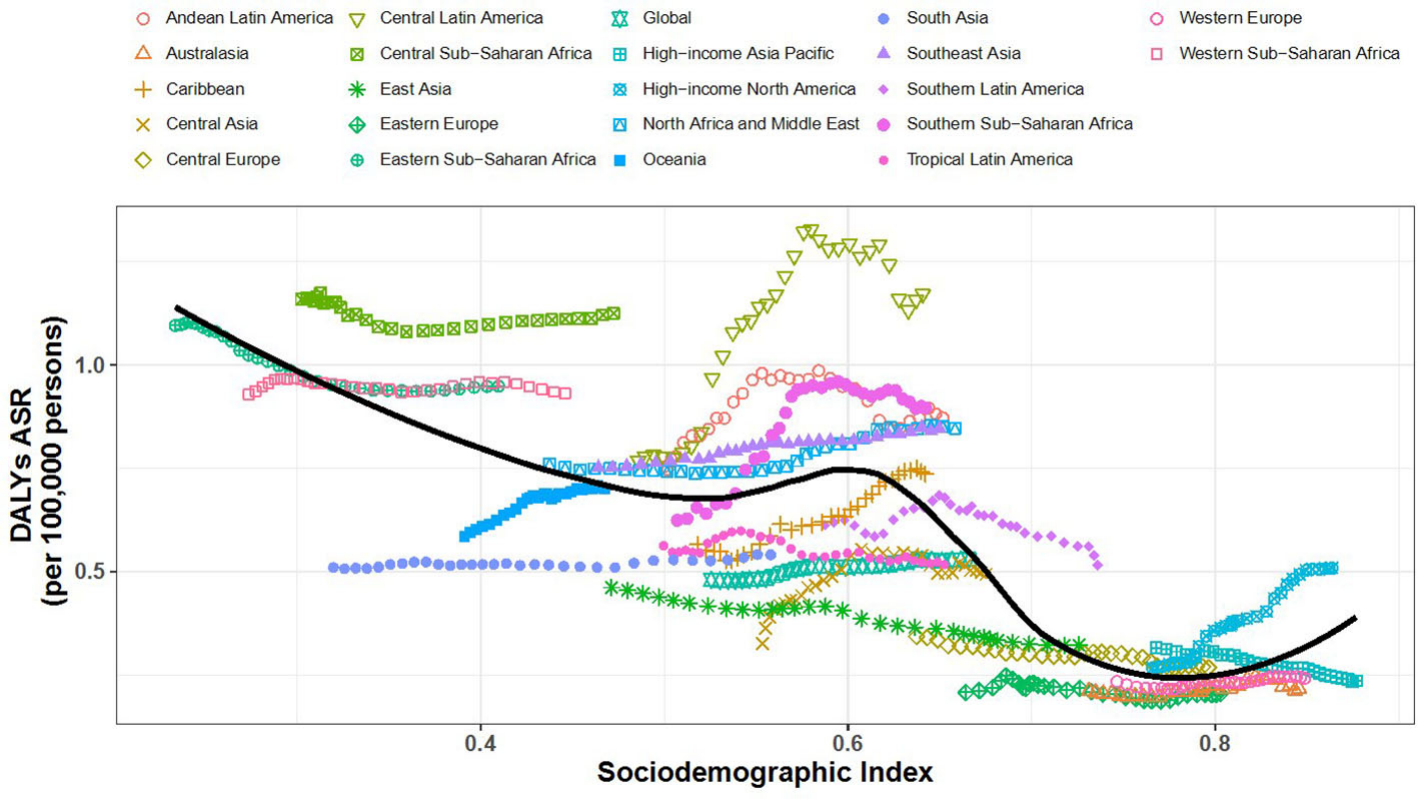
Relationship Between SDI and CKD-Related DALYs by Region.

**Figure 7:**
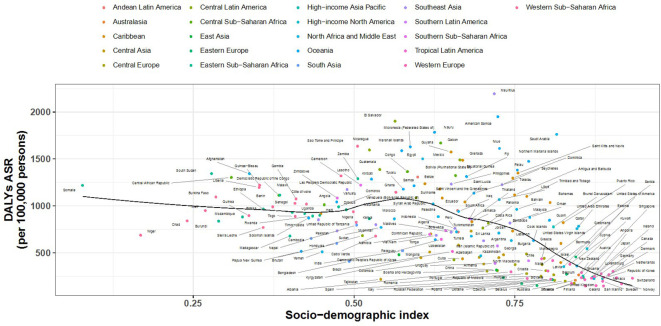
Country-Level SDI and CKD-Related DALYs Analysis.

**Figures 6**, **7** show the association between the Socio-Demographic Index (SDI) and CKD-related DALYs across various regions and countries in 2021. Overall, as the SDI increases, CKD burden initially declines but then rises again around an SDI value of 0.6. Some regions exhibit a reverse V-shaped pattern, where DALY rates increase with SDI until a certain point before decreasing, particularly in Central America, Southern sub-Saharan Africa, and Western Europe. Certain areas, such as Central sub-Saharan Africa and Central America, have higher-than-expected CKD burdens, while South Asia and Tropical Latin America show lower-than-expected burdens.

### 3.5 Risk Factor Analysis

**Figure 8:**
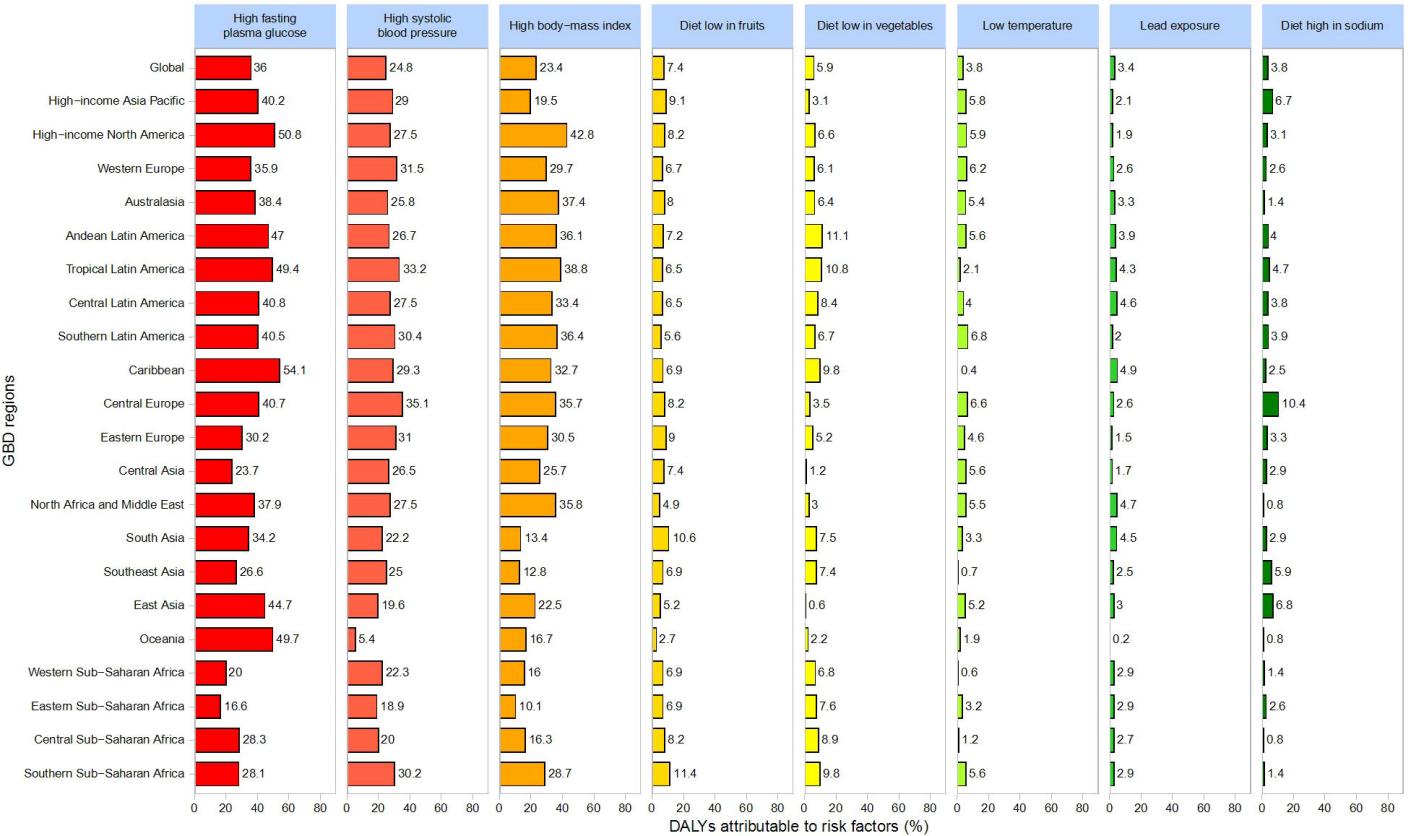
Major Health Risk Factors for CKD and Their Contribution to DALYs.

**Figure 8** provides an overview of the primary health risk factors associated with CKD and their relative contributions to DALYs. Globally, the key risk factors include high fasting plasma glucose (36%), high systolic blood pressure (24.8%), high body mass index (23.4%), low fruit intake (7.4%), low vegetable intake (5.9%), low temperature (3.8%), lead exposure (3.4%), and high sodium intake (3.8%).

Regionally, high fasting plasma glucose contributes most significantly to DALYs, especially in the Caribbean (54.1%), High-Income North America (50.8%), Oceania (49.7%), and Tropical Latin America (49.4%). High systolic blood pressure is particularly impactful in Central Europe (35.1%), followed by Tropical Latin America (33.2%) and Western Europe (31.5%). High BMI exerts the greatest influence in High-Income North America (42.8%), Tropical Latin America (38.8%), and Oceania (37.4%). Additionally, low fruit intake plays a notably significant role in the Middle East (15%) and East Asia (12%).

## 4 Discussion

This study provides new insights into the global and regional characteristics of chronic kidney disease (CKD) from 1990 to 2021, highlighting important trends and key determinants that can inform future prevention and control strategies. Although the age-standardized prevalence rate (ASPR) of CKD declined slightly over the past 30 years (-0.8%), differing from some previous findings (2), mortality and disability-adjusted life years (DALYs) remain substantially high worldwide. In particular, low Socio-Demographic Index (SDI) regions—such as Eastern, Central, and Southern sub-Saharan Africa and South Asia—continue to experience a growing DALYs burden, underscoring persistent shortcomings in healthcare resource allocation, early disease screening, and timely intervention (17).

Consistent with existing literature, our results confirm that CKD burdens are heaviest in low-SDI regions (18). Notably, our refined analyses reveal persistently elevated ASPR and DALYs in Eastern, Central, and Southern sub-Saharan Africa, providing more targeted data to support regional disease management efforts. Furthermore, our findings regarding age- and sex-related differences in CKD prevalence align with prior studies indicating higher rates among females (19). Such disparities may be linked to physiological factors, including hormonal changes (20, 21), as well as sociocultural elements like healthcare accessibility and health-seeking behaviors.

### 4.1 Regional impacts of health risk factors

This study identifies high fasting plasma glucose, high systolic blood pressure, and high body mass index (BMI) as primary global risk factors for CKD, yet their contributions vary by region. For example, high fasting plasma glucose accounts for 36% of global DALYs and is particularly influential in the Caribbean and High-Income North America, while high BMI contributes notably to the CKD burden in High-Income North America (42.8%). Central Europe and Tropical Latin America must pay closer attention to the impact of high systolic blood pressure on CKD outcomes. Such geographic variations highlight the need for region-specific risk factor mitigation strategies.

### 4.2 Policy implications and intervention priorities

Given the regional disparities in health risk factors, it is imperative to design targeted, region-specific interventions (22–24). High-income regions should focus on addressing obesity and aging populations, whereas low-SDI areas must prioritize strengthening healthcare infrastructure (25, 26) and improving screening and control of diabetes and hypertension (27, 28). For regions such as sub-Saharan Africa and South Asia, where the DALY burden continues to rise, international collaboration and strategic resource allocation are crucial. Enhancing the implementation of public health policies and mitigating environmental risk factors will be essential for reversing these trends (29, 30).

### 4.3 Limitations

This study has several limitations. First, the analyses rely substantially on modeled estimates, introducing a degree of uncertainty, particularly for data-scarce regions (31). Second, as our assessment is based on existing public databases, the absence of high-quality data for some countries or regions may have limited the comprehensiveness of our evaluation. Additionally, variations in the definitions or measurement methods for certain risk factors could introduce bias and affect the interpretation of our findings. Moreover, the Global Burden of Disease dataset does not explicitly account for certain unmeasured confounders such as healthcare access disparities, cultural practices, or genetic susceptibility, which may partly explain regional differences.

## 5 Conclusion

This study reveals that, despite a slight global decline in the CKD age-standardized prevalence rate (-0.8%) from 1990 to 2021, mortality and DALYs significantly increased in low-SDI regions (e.g., Eastern, Central, and Southern sub-Saharan Africa and South Asia), underscoring limited healthcare resources and inadequate chronic disease management. High fasting plasma glucose, high systolic blood pressure, and high BMI emerged as key risk factors with varying regional impacts. Female prevalence surpasses male rates after age 30 and remains higher. These findings provide a scientific basis for targeted interventions and enhanced international collaboration to reduce the global CKD burden.

## Data Availability

The original contributions presented in the study are included in the article/**Supplementary Material**. Further inquiries can be directed to the corresponding author/s.

## Supplementary material

Supplementary Table 1 Prevalent cases of chronic kidney disease in 1990 and 2021 and the percentage change in the age-standardized rates (ASRs) per 100,000, by location.

Supplementary Table 2 Deaths due to chronic kidney disease from 1990 to 2021 and the percentage change in the age-standardized mortality rates (ASMRs) per 100,000, by location.

Supplementary Table 3 DALYs due to chronic kidney disease from 1990 to 2021 and the percentage change in the age-standardized rates (ASRs) per 100,000, by location.

## References

[1] Francis A Harhay MN Ong A Tummalapalli SL Ortiz A Fogo AB. Chronic kidney disease and the global public health agenda: an international consensus. Nat Rev Nephrol. (2024) 3:1–3. doi: 10.1038/s41581-024-00820-6 38570631

[2] Bikbov B Purcell CA Levey AS Smith M Abdoli A Abebe M. Global, regional, and national burden of chronic kidney disease, 1990–2017: a systematic analysis for the Global Burden of Disease Study 2017. Lancet. (2020) 395:709–33. doi: 10.1016/S0140-6736(20)30045-3 PMC7049905 32061315

[3] Anand S Zheng Y Montez-Rath ME Wei WJ Perico N Carminati S. Do attributes of persons with chronic kidney disease differ in low-income and middle-income countries compared with high-income countries? Evidence from population-based data in six countries. BMJ Global Health. (2017) 2:e000453. doi: 10.1136/bmjgh-2017-000453 PMC5640036 29071132

[4] Ruiz-Ortega M Rayego-Mateos S Lamas S Ortiz A Rodrigues-Diez RR. Targeting the progression of chronic kidney disease. Nat Rev Nephrol. (2020) 16:269–88. doi: 10.1038/s41581-019-0248-y 32060481

[5] Lakkis JI Weir MR. Obesity and kidney disease. Prog Cardiovasc Dis. (2018) 61:157–67. doi: 10.1016/j.pcad.2018.07.005 29981350

[6] Thomas MC Cooper ME Zimmet P. Changing epidemiology of type 2 diabetes mellitus and associated chronic kidney disease. Nat Rev Nephrol. (2016) 12:73–81. doi: 10.1038/nrneph.2015.173 26553517

[7] Rossing P Hansen TW Kümler T. Cardiovascular and non-renal complications of chronic kidney disease: managing risk. Diabetes Obes Metab. (2024) 26 Suppl 6:13–21. doi: 10.1111/dom.15747 38982587

[8] Komenda P Ferguson TW Macdonald K Rigatto C Koolage C Sood MM. Cost-effectiveness of primary screening for CKD: a systematic review. Am J Kidney Dis. (2014) 63:789–97. doi: 10.1053/j.ajkd.2013.12.012 24529536

[9] Folkerts K Petruski-Ivleva N Comerford E Blankenburg M Evers T Gay A. Adherence to chronic kidney disease screening guidelines among patients with type 2 diabetes in a US administrative claims database. Mayo Clin Proc. (2021) 96:975–86. doi: 10.1016/j.mayocp.2020.07.037 33722396

[10] Cockwell P Fisher LA. The global burden of chronic kidney disease. Lancet. (2020) 395:662–4. doi: 10.1016/S0140-6736(19)32977-0 32061314

[11] Romagnani P Remuzzi G Glassock R Levin A Jager KJ Tonelli M. Chronic kidney disease. Nat Rev Dis Primers. (2017) 3:1–24. doi: 10.1038/nrdp.2017.88 29168475

[12] Brennan E Kantharidis P Cooper ME Godson C. Pro-resolving lipid mediators: regulators of inflammation, metabolism and kidney function. Nat Rev Nephrol. (2021) 17:725–39. doi: 10.1038/s41581-021-00454-y PMC8287849 34282342

[13] Lao XQ Bo Y Chen D Zhang K Szeto CC. Environmental pollution to kidney disease: an updated review of current knowledge and future directions. Kidney Int. (2024) 106:214–25. doi: 10.1016/j.kint.2024.04.021 38797324

[14] Zang Y Devleesschauwer B Bolger PM Goodman E Gibb HJ. Global burden of late-stage chronic kidney disease resulting from dietary exposure to cadmium, 2015. Environ Res. (2019) 169:72–8. doi: 10.1016/j.envres.2018.10.005 30419431

[15] Schumacher AE Kyu HH Kisa A GBD 2021 Demographics Collaborators. Global age-sex-specific mortality, life expectancy, and population estimates in 204 countries and territories and 811 subnational locations, 1950–2021, and the impact of the COVID-19 pandemic: a comprehensive demographic analysis for the Global Burden of Disease Study 2021. Lancet. (2024) 403:1989–2056. doi: 10.1016/S0140-6736(24)00476-8 PMC11126395 38484753

[16] Institute for Health Metrics and Evaluation. Global health data exchange(2024). Available online at: https://ghdx.healthdata.org/ (Accessed November 17, 2024).

[17] Campbell ZC Dawson JK Kirkendall SM McCaffery KJ Jansen J Campbell KL. Interventions for improving health literacy in people with chronic kidney disease. Cochrane Database Syst Rev. (2022) 12:CD012026. doi: 10.1002/14651858.CD012026.pub2 36472416 PMC9724196

[18] Garcia-Garcia G Jha V. Nephrology in the developing world: Chronic kidney disease in disadvantaged populations. Nat Rev Nephrol. (2015) 11:128. doi: 10.1038/nrneph.2015.4 25629510

[19] Kovesdy CP. Epidemiology of chronic kidney disease: an update 2022. Kidney Int Suppl. (2022) 12:7–11. doi: 10.1016/j.kisu.2021.11.003 PMC9073222 35529086

[20] Levey AS Coresh J. Chronic kidney disease. Lancet. (2012) 379:165–80. doi: 10.1016/S0140-6736(11)60178-5 21840587

[21] Ashuntantang GE Garovic VD Heilberg IP Lightstone L. Kidneys and women’s health: key challenges and considerations. Nat Rev Nephrol. (2018) 14:203–10. doi: 10.1038/nrneph.2017.188 29380816

[22] Declèves AE Sharma K. Novel targets of antifibrotic and anti-inflammatory treatment in CKD. Nat Rev Nephrol. (2014) 10:257–67. doi: 10.1038/nrneph.2014.31 PMC6287621 24662433

[23] Rabelink TJ Little MH. Stromal cells in tissue homeostasis: balancing regeneration and fibrosis. Nat Rev Nephrol. (2013) 9:747–53. doi: 10.1038/nrneph.2013.152 23938596

[24] Luyckx VA Tuttle KR Garcia-Garcia G Gharbi MB Heerspink HJL Johnson DW. Reducing major risk factors for chronic kidney disease. Kidney Int Suppl. (2017) 7:71–87. doi: 10.1016/j.kisu.2017.07.003 PMC6341126 30675422

[25] Webster AC Nagler EV Morton RL Masson P. Chronic kidney disease. Lancet. (2017) 389:1238–52. doi: 10.1016/S0140-6736(16)32064-5 27887750

[26] Câmara NOS Iseki K Kramer H Liu ZH Sharma K. Kidney disease and obesity: epidemiology, mechanisms and treatment. Nat Rev Nephrol. (2017) 13:181–90. doi: 10.1038/nrneph.2016.191 28090083

[27] Selby NM Taal MW. What every clinician needs to know about chronic kidney disease: Detection, classification and epidemiology. Diabetes Obes Metab. (2024) 26 Suppl 6:3–12. doi: 10.1111/dom.15683 38804058

[28] Lousa I Reis F Beirão I Alves R Belo L Santos-Silva A. New potential biomarkers for chronic kidney disease management—A review of the literature. Int J Mol Sci. (2020) 22:43. doi: 10.3390/ijms22010043 33375198 PMC7793089

[29] Pena MJ Stenvinkel P Kretzler M Adu D Agarwal SK Coresh J. Strategies to improve monitoring disease progression, assessing cardiovascular risk, and defining prognostic biomarkers in chronic kidney disease. Kidney Int Suppl. (2017) 7:107–13. doi: 10.1016/j.kisu.2017.07.005 PMC6341006 30675424

[30] Coresh J Hu JR Bello AK Feldman HI Fogo AB Ganji MR. Action plan for determining and monitoring the prevalence of chronic kidney disease. Kidney Int Suppl. (2017) 7:63–70. doi: 10.1016/j.kisu.2017.07.002 PMC6341010 30675421

[31] Sukumar Vellakkal SV Millett C Basu S Zaky Khan ZK Aitsi-Selmi A Stuckler D. Are estimates of socioeconomic inequalities in chronic disease artefactually narrowed by self-reported measures of prevalence in low-income and middle-income countries? Findings from the WHO-SAGE survey. J Epidemiol Community Health. (2015) 69:218–25. doi: 10.1136/jech-2014-204621 PMC4345525 25550454

