# Supplementary material for: Global burden of chronic kidney disease and its attributable risk factors (1990-2021): an analysis based on the global burden of disease study
Source: Front Endocrinol (Lausanne). 2025 Jul 3;16:1563246. doi: 10.3389/fendo.2025.1563246 (PMC12267038; doi:10.3389/fendo.2025.1563246)
Supplement: Supplementary Table 1 — Prevalent cases of chronic kidney disease in 1990 and 2021 and the percentage change in the age-standardized rates (ASRs) per 100,000, by location. [file DataSheet1.pdf]

Table S1： Prevalent cases of chronic kidney disease in 1990 and 2021 and the percentage change in the age-standardised rates (ASRs) per 100,000, by location

	1990		2021		Percentage change in the ASRs per 100,000
	No (95% UI)	ASRs per 100,000 (95% UI)	No (95% UI)	ASRs per 100,000 (95% UI)	
Global	350962674 (326973785,376155723)	8072.8 (7560.4,8634.1)	673722703 (629095119,722364096)	8006 (7482.1,8575.6)	-0.8 (-1.9,0.1)
High-income North America	25125238 (23432071,26893288)	7401.1 (6920.4,7900.3)	42488578 (39404535,45165702)	7434.7 (6959.3,7911.4)	0.5 (-0.6,1.8)
Canada	2229477 (2055472,2415616)	7026.4 (6480.6,7603.7)	3809329 (3540568,4060952)	6524.4 (6091.5,7000.7)	-7.1 (-13.5,-2.3)
Greenland	2958 (2729,3212)	6970.7 (6515.6,7414.6)	4412 (4094,4732)	6988.2 (6509.1,7491.4)	0.3 (-2.6,3.1)
United States of America	22892228 (21306718,24521743)	7438.3 (6947,7942.8)	38674171 (35778672,41193631)	7550.8 (7050.6,8067.4)	1.5 (0.3,2.7)
Australasia	1399739 (1315064,1481429)	6084.4 (5719.4,6456.3)	2810777 (2637789,2995329)	5910 (5548.2,6301.6)	-2.9 (-5.7,0.1)
Australia	1165447 (1094777,1238029)	6076.8 (5714.8,6452.5)	2353593 (2211156,2523412)	5877.9 (5525.9,6272.1)	-3.3 (-6.5,0.4)
New Zealand	234292 (217327,250752	6128.4 (5708.2,6546.6)	457184 (423466,488353	6072.6 (5649.2,6499)	-0.9 (-3.3,1.6)
High-income Asia Pacific	16878162 (15709632,18083496)	8566.1 (7979.5,9169.5)	29276226 (27336162,31020670)	7920.7 (7393.7,8457.4)	-7.5 (-8.5,-6.6)
Brunei Darussalam	15220 (13998,16528)	9497.7 (8852.2,10130.5	39451 (36520,42780)	9461.9 (8796.6,10168.9	-0.4 (-3.3,2.4)
Japan	13889911 (12903717,14885294)	8744.8 (8150.4,9364.1)	22877631 (21341971,24294034)	8264.3 (7717.7,8855.7)	-5.5 (-6.2,-4.7)
Singapore	269496 (247876,292201	9889.3 (9177.1,10490.2	738749 (680520,792459	9230 (8547.7,9877.1)	-6.7 (-9.9,-3.5)
Republic of Korea	2703535 (2505415,2935211)	7789.4 (7280,8381.1)	5620395 (5254764,6008210)	6872.2 (6417,7364.4)	-11.8 (-15.2,-8.6)
Western Europe	29093594 (27230515,30911868)	5469.9 (5132.6,5799.4)	41591466 (39070950,43910754)	5226.2 (4924.4,5544.2)	-4.5 (-6.2,-3)
Andorra	3305 (3059,3549)	5604.9 (5216.1,5996.5)	7623 (7073,8129)	5535 (5147.9,5919.3)	-1.2 (-4.2,1.4)
Austria	611202 (567555,653621	5590.9 (5205.7,5978.5)	892225 (838208,953902	5671 (5326.4,6084.5)	1.4 (-2.8,5.8)
Belgium	808609 (750019,867640)	5722.1 (5342.5,6109.5)	1120459 (1051828,1196504)	5633.2 (5305.5,6009.4)	-1.6 (-5.9,2.8)
Cyprus	47988 (44580,51332)	6009 (5608.3,6395)	112031 (104079,120582	5817.6 (5430.8,6247.9)	-3.2 (-6,-0.4)

Denmark	415234 (386838,442513	5589.5 (5229.3,5953.9)	564571 (524056,607172	5607 (5237.4,6009.8)	0.3 (-3.9,4.8)
Finland	368157 (345612,392962	5556.6 (5209.3,5940.6)	555910 (513129,595333	5349.2 (4968.6,5714.9)	-3.7 (-6.7,-0.7)
France	3209806 (2980224,3426824)	4202.2 (3898.7,4488.2)	5214795 (4858423,5614715)	4368.8 (4085.6,4698.7)	4 (-0.3,8.5)
Germany	6261066 (5866849,6644060)	5349.6 (5017.4,5675)	8931944 (8467049,9441979)	5278.4 (4985.5,5622.2)	-1.3 (-5.3,2.1)
Greece	798820 (740666,853941)	5762.3 (5361.4,6162.9)	1143247 (1066167,1220356)	5587.8 (5236.3,5992.8)	-3 (-6.9,0.8)
Iceland	13378 (12516,14287)	4739 (4420.2,5063.2)	23128 (21625,24673)	4528.9 (4231,4840.2)	-4.4 (-7.3,-1.6)
Ireland	261985 (241344,282388	6681.7 (6202.5,7184.3)	465353 (440528,488132	6594.1 (6237.5,6952)	-1.3 (-6.2,4.1)
Israel	288923 (270285,308809	6007.8 (5618.9,6420.3)	674873 (630913,722204	5866.8 (5487.9,6273.7)	-2.3 (-6.3,1.9)
Italy	4457622 (4142484,4769497)	5591.9 (5235.5,5978.8)	5921997 (5501809,6294811)	5061.3 (4746.2,5410.5)	-9.5 (-11.1,-7.5)
Luxembourg	30243 (27852,32537)	5937.6 (5491.4,6400.1)	55107 (50921,59381)	5775.6 (5340.1,6220.8)	-2.7 (-6.4,0.6)
Malta	24308 (22689,26237)	5881.7 (5499.5,6324.9)	46498 (43037,49500)	5665.8 (5292.2,6053.1)	-3.7 (-6.7,-0.6)
Monaco	3295 (3048,3530)	5539.9 (5150.2,5916.3)	4350 (4042,4640)	5497.2 (5141.2,5862.9)	-0.8 (-3.8,2.1)
Netherlands	1147619 (1073580,1226033)	6067.4 (5675.9,6498.2)	1745668 (1634215,1877107)	6048.2 (5674.3,6481.1)	-0.3 (-3.2,2.9)
Norway	314586 (291897,335963	5207.1 (4866.3,5562)	469377 (437256,500382	5534.4 (5167.8,5922.5)	6.3 (5.1,7.5)
Portugal	641583 (596339,685707)	5124.5 (4804.7,5501.1)	941606 (850524,1011350)	4777 (4420.4,5132.3)	-6.8 (-12.1,-3.6)
San Marino	1771 (1651,1899)	5453.5 (5097.1,5832.3)	3397 (3165,3648)	5392.6 (5034.8,5768.8)	-1.1 (-3.7,1.6)
Spain	2768974 (2588950,2935877)	5464.9 (5131.1,5781.9)	4288430 (3948305,4576051)	5022.8 (4691,5348.8)	-8.1 (-14.4,-4.4)
Sweden	821047 (759582,881515)	6415.6 (5943,6890.7)	1121779 (1040388,1207272)	6424 (5973.5,6887.8)	0.1 (-2.1,2.4)
Switzerland	639460 (600031,681319)	6582.8 (6179,7026.2)	995630 (934109,1061548)	6354 (5957.2,6788.6)	-3.5 (-6.5,-0.2)
United Kingdom	5130703 (4779037,5480493)	6119.8 (5722.5,6520.2)	6254851 (5837368,6635678)	5449.1 (5106.2,5788.7)	-11 (-12.2,-9.7)
Southern Latin America	2695843 (2510441,2883801)	5784.4 (5397.3,6182.9)	4860631 (4530777,5214539)	5970.4 (5543.1,6415)	3.2 (0.7,6.1)

Argentina	1774583 (1648138,1900776)	5585.8 (5197.5,5980)	3114327 (2893145,3345866)	5876.1 (5442.4,6323.9)	5.2 (1.8,9.7)
Chile	699136 (650457,755320)	6211 (5786.9,6653.9)	1444161 (1343232,1545985)	6106.6 (5659.6,6545.9)	-1.7 (-4.8,1.4)
Uruguay	221996 (206709,237075	6192.6 (5767.9,6613.6)	301874 (280956,322913	6282.8 (5818,6738.2)	1.5 (-2,4.6)
Eastern Europe	24173788 (22444048,26062971)	9327.5 (8680,10050.1)	27312843 (25403078,29335270)	9266.3 (8619.4,9989.5)	-0.7 (-1.5,0.2)
Belarus	1162192 (1079543,1248012)	9654.1 (8993.2,10364.9)	1314740 (1220742,1405904)	9704.3 (9000.4,10430.5)	0.5 (-2.8,3.2)
Estonia	181645 (168130,196037	9678.7 (8968.1,10435.8	206581 (192387,221597	9660.9 (9006.3,10389.7	-0.2 (-2.3,2)
Latvia	313358 (290679,336853	9660.9 (8967.6,10391.3	303626 (282328,323377	9785.8 (9110.5,10514.3	1.3 (-1.1,3.8)
Lithuania	414295 (384463,446122	9790.2 (9086.7,10563.5	441211 (411206,472545	9837.2 (9134.9,10590)	0.5 (-3,3.9)
Republic of Moldova	469578 (435381,501649)	10729.3 (10014.3,11456.7)	596118 (553806,636138)	11293.7 (10519.6,12048.1)	5.3 (1.9,8.5)
Russian Federation	15387961 (14274967,16655381)	9062.2 (8429.2,9771.4)	18056081 (16744178,19455656)	8973.8 (8345.5,9704.8)	-1 (-1.7,-0.2)
Ukraine	6244758 (5793184,6760948)	9772.5 (9071.5,10538.3)	6394485 (5969352,6867844)	9814.1 (9113.9,10548.5)	0.4 (-2.1,2.8)
Central Europe	8932587 (8363724,9525233)	6368.6 (5978,6772)	11481266 (10766159,12145281)	6205.2 (5841,6596.2)	-2.6 (-4.1,-1)
Albania	152351 (142462,163786	6243.7 (5830.9,6667.4)	230609 (214471,246180	6154 (5711.8,6544.9)	-1.4 (-4.3,1.4)
Bosnia and Herzegovina	260939 (242972,279575	6132.4 (5735.3,6544.3)	323305 (300335,346499	6233.5 (5827.2,6665.1)	1.6 (-1.3,4.6)
Bulgaria	673785 (626288,720194	6187.8 (5773,6611.1)	726001 (674875,778060	6282.1 (5866.9,6704.4)	1.5 (-3.2,6.1)
Croatia	352794 (326765,378976	6188.9 (5755.5,6629)	441010 (407740,471831	6109.1 (5670,6576.3)	-1.3 (-4.8,2.3)
Czechia	777458 (723745,829773)	6159.6 (5756.9,6580.7)	1033665 (960274,1099534)	5875 (5479.3,6268.4)	-4.6 (-7.4,-1.4)
Hungary	816867 (760112,874417)	6193.2 (5788.2,6630.4)	965275 (898080,1028450)	5992.9 (5610.7,6393.6)	-3.2 (-6.3,-0.5)
Montenegro	40300 (37650,42973)	6416 (6010.8,6835.5)	54152 (50266,57833)	6415 (5962.1,6863.8)	0 (-2.6,2.6)
North Macedonia	140331 (130960,149494	7386 (6902,7876.8)	220103 (205048,234493	7377.4 (6929.9,7832.2)	-0.1 (-3.1,3.2)
Poland	2866015 (2680925,3059816)	6850.9 (6418.5,7310.9)	3872502 (3620089,4134821)	6415.3 (6012.6,6855.3)	-6.4 (-7.8,-4.8)

Romania	1605525 (1495525,1715394)	6180.4 (5775.4,6591.1)	1993675 (1890808,2098153)	6300.7 (5964.6,6652.5)	1.9 (-2.1,5.8)
Serbia	603591 (559791,648669	5667.2 (5283.7,6069)	753941 (699986,811166	5593.3 (5189.2,6041)	-1.3 (-4.2,1.6)
Slovakia	357483 (334696,382908	6221.6 (5843.7,6645)	486371 (454761,518472	5960 (5575.9,6352.7)	-4.2 (-7.1,-1.3)
Slovenia	142299 (133101,152114	6083.6 (5696.5,6507.4)	213545 (199557,227477	5968.2 (5539.4,6369.4)	-1.9 (-5.4,1.8)
Central Asia	5534240 (5182702,5905060)	10649.1 (10005.7,11309.3)	9356434 (8704146,9969948)	10698.2 (10022.9,11348.1)	0.5 (-1.3,2.1)
Armenia	300635 (278865,323273	10265.9 (9580.8,10974)	404070 (378068,430935	10245 (9598,10928)	-0.2 (-4,3.9)
Azerbaijan	593465 (554684,636804)	10594.8 (9927.5,11288.4)	1146469 (1073053,1221894)	10696 (10019,11338.2)	1 (-2,4.4)
Georgia	632971 (590879,679007	10617.5 (9919.4,11342.7	544204 (506648,579897	10551.1 (9851.3,11224)	-0.6 (-4.5,3.5)
Kazakhstan	1471284 (1368347,1573180)	10730.4 (10056.3,11412)	1952569 (1812225,2090896)	10662.4 (9928.1,11394.4)	-0.6 (-3.2,1.9)
Kyrgyzstan	349912 (326466,372023	10650.2 (9986.3,11306.1	554675 (518240,592588	10147.7 (9422.6,10780.3	-4.7 (-7.7,-1.7)
Mongolia	146040 (136801,156132)	11239.6 (10569.3,11958.8)	287978 (267255,309250)	10667.7 (9937.1,11381.2)	-5.1 (-8.1,-1.9)
Tajikistan	322282 (300325,346451	9924.7 (9264.6,10630.8	708588 (659872,761599	9851.2 (9246.7,10479)	-0.7 (-3.6,2.4)
Turkmenistan	254957 (238341,273926)	11011.1 (10342.1,11708.9)	484074 (452902,515793)	10909.7 (10221.7,11599.9)	-0.9 (-4.6,2.9)
Uzbekistan	1462693 (1370470,1565221)	10757.8 (10084.1,11467.1)	3273806 (3046385,3512972)	11069.1 (10338.8,11793.6)	2.9 (-0.1,6.1)
Central Latin America	8628113 (8065183,9238847)	8477 (7931,9062.8)	22101801 (20701763,23422153)	8642.9 (8089.3,9163.7)	2 (0.1,3.9)
Colombia	1697055 (1583361,1826064)	7912.4 (7376.2,8461.8)	4222423 (3939788,4478779)	7695.6 (7194,8158.8)	-2.7 (-6.5,1.3)
Costa Rica	197345 (183956,211905	9636.7 (8996.2,10298.8	517788 (484174,550601	9532.1 (8909.8,10127.1	-1.1 (-5.4,4.4)
El Salvador	239034 (222018,257099	6892.2 (6378.2,7415.7)	460009 (431238,489587	7296.1 (6841.6,7771)	5.9 (1.3,10.5)
Guatemala	342544 (318619,365583)	8065.5 (7574.1,8638.4)	1096863 (1023152,1176256)	8857.7 (8275.1,9478.3)	9.8 (6,13.6)
Honduras	209413 (194859,225041	8405.3 (7844.1,8968.8)	633449 (591340,678996	8613 (8025.9,9216)	2.5 (-0.9,6)
Mexico	4594717 (4287702,4924515)	8781.5 (8206.6,9393.8)	11780174 (11009027,12569139)	9052.2 (8453.8,9672.7)	3.1 (1.5,4.6)
Nicaragua	190029 (177519,203548	9675.2 (9075.4,10313.2	575347 (537918,614144	10511.2 (9794.3,11157.8	8.6 (3.6,14.9)

Panama	142393 (132809,153036	8261.6 (7709.3,8864.8)	373196 (347346,398963	8453.5 (7857.2,9031.7)	2.3 (-2.4,6.4)
Venezuela （Bolivarian Republic of)	1015584 (943619,1092889)	8453 (7868.5,9056.9)	2442552 (2275365,2599385)	8317.3 (7756.4,8846.8)	-1.6 (-6,3.5)
Andean Latin America	1465459 (1365798,1575973)	5766.6 (5377.8,6181)	3743589 (3478926,4028408)	5946.1 (5524.8,6371.5)	3.1 (0.5,5.2)
Bolivia (Plurinational State of)	242900 (225810,260389	6093.3 (5681.1,6496.9)	628320 (584306,676494	6177.1 (5740.2,6632.4)	1.4 (-1.2,4)
Ecuador	392453 (366304,421830)	5893.4 (5492,6356.8)	1073132 (996177,1149397)	6249.3 (5811.7,6689.2)	6 (2.2,9.9)
Peru	830105 (773577,894482)	5621.6 (5237.5,6016)	2042137 (1897519,2195297)	5732.7 (5325.1,6154.3)	2 (-1.3,4.6)
Caribbean	1867236 (1741663,2000227)	6480.2 (6037.6,6925.8)	3468705 (3235452,3720720)	6633 (6181.3,7112.4)	2.4 (0.7,4)
Antigua and Barbuda	3729 (3474,3994)	6700.1 (6253.5,7179.1)	7270 (6762,7815)	6956.3 (6479.9,7455.7)	3.8 (0.8,6.7)
Barbados	17997 (16818,19169)	6459.9 (6051.7,6909.2)	28988 (27004,31010)	6676.8 (6215.3,7161.7)	3.4 (0.7,6)
Belize	7609 (7071,8133)	6629.9 (6188.8,7130.9)	24923 (23248,26780)	6996.9 (6519.9,7476.1)	5.5 (1.7,9.4)
Bermuda	4041 (3755,4358)	6209.7 (5792.5,6656.9)	6571 (6087,7048)	6114.2 (5679.3,6583.5)	-1.5 (-4.2,1.2)
Bahamas	12715 (11806,13734)	6542.7 (6098.9,7002.9)	28177 (26096,30221)	6759.3 (6258.6,7239.1)	3.3 (0.7,6.1)
Cuba	670575 (622916,717810)	6258.5 (5824.6,6706.2)	1023168 (949443,1092062)	6157.3 (5728.3,6614.3)	-1.6 (-4.8,1.5)
Dominica	4263 (3961,4572)	6991.4 (6509.2,7479.9)	5745 (5329,6134)	7303.9 (6813,7818.6)	4.5 (1.7,7.2)
Dominican Republic	299732 (277339,324408	6357.9 (5927.6,6822.6)	710332 (660993,766986	6688.8 (6211.1,7193.2)	5.2 (2.4,8.3)
Grenada	5042 (4684,5387)	6983.1 (6481.8,7464)	8182 (7614,8784)	7279.5 (6800.4,7818.7)	4.2 (1.5,7.1)
Guyana	34192 (31637,36857)	6803.9 (6325.9,7280.5)	48899 (45521,52822)	7062.5 (6579,7608.2)	3.8 (0.6,7)
Haiti	274957 (254724,296317	6959.2 (6473,7441.6)	662956 (613153,717829	7123.7 (6614.8,7647.9)	2.4 (0.1,4.4)
Jamaica	122884 (115047,131333	6456.9 (6045.3,6921.3)	209554 (194578,224704	6708.1 (6219,7188.8)	3.9 (0.8,7)
Puerto Rico	241664 (224964,259466	6681.7 (6222.4,7166.2)	380144 (353031,405700	6833 (6356.4,7333.3)	2.3 (-0.9,5.2)
Saint Kitts and Nevis	2582 (2409,2774)	7034.2 (6570.2,7551.8)	4826 (4470,5192)	6962.4 (6470,7455.7)	-1 (-3.8,1.5)
Saint Lucia	6820 (6312,7305)	6901.6 (6410.1,7371.5)	15679 (14634,16864)	6954.1 (6489.8,7468.2)	0.8 (-2,3.3)
Saint Vincent and the Grenadines	5218 (4816,5601)	6544.4 (6043.5,7001.3)	9229 (8590,9910)	6902.6 (6435.8,7413.5)	5.5 (2.5,8.4)
Suriname	19697 (18356,21176)	6671.6 (6215.3,7151.6)	42747 (39950,45625)	6878.3 (6435.8,7347.1)	3.1 (0,6.1)

Trinidad and Tobago	64179 (59428,68941)	6674.1 (6204.2,7119.3)	124244 (115206,133282	7009.5 (6525,7524.9)	5 (1.8,8.2)
United States Virgin Islands	6113 (5650,6602)	6554.3 (6091.8,7043.7)	9684 (8946,10346)	6791.6 (6302.6,7286)	3.6 (1.2,6.2)
Tropical Latin America	8408163 (7820461,9015639)	7763 (7240.7,8281.3)	19295703 (18022781,20680042)	7576.2 (7081,8107.5)	-2.4 (-3.4,-1.4)
Brazil	8215420 (7640129,8812335)	7772.7 (7248.7,8291.2)	18814692 (17566365,20174535)	7576.6 (7078.5,8111.2)	-2.5 (-3.6,-1.5)
Paraguay	192742 (179980,206867	7374.3 (6876.7,7891.9)	481010 (448476,516329	7561.4 (7051.6,8091.3)	2.5 (-0.8,6.7)
East Asia	69797869 (64858255,75190145)	7083.6 (6610.1,7593.5)	122849232 (113607491,132118615)	6258.1 (5823.1,6729.8)	-11.7 (-13.5,-10.2)
China	67398525 (62608781,72614585)	7091.9 (6614.3,7603.1)	118403911 (109388671,127484286)	6249.4 (5812.4,6720.2)	-11.9 (-13.7,-10.4)
Democratic People's Republic of Korea	1150078 (1067243,1233674)	6571.8 (6117.4,7043.5)	2027239 (1883443,2173423)	6390.8 (5955.9,6816.8)	-2.8 (-5.4,0)
Taiwan (Province of China)	1249266 (1163816,1344723)	7155.4 (6728.1,7647.1)	2418082 (2270339,2579826)	6571.2 (6152.2,7040.8)	-8.2 (-11,-5.4)
Southeast Asia	33768821 (31068003,36689892)	10225.2 (9474,11022.1)	73561437 (67779796,79733436)	10474.6 (9718.9,11301.7)	2.4 (1.6,3.2)
Cambodia	566875 (518696,615039)	9455 (8749.4,10222.8)	1378217 (1266270,1503301)	9453 (8751.4,10231.3)	0 (-2.2,2.5)
Indonesia	13599739 (12496422,14810294)	10447.7 (9661.9,11264.5)	29413896 (26931317,32010165)	10740.3 (9942.1,11598.8)	2.8 (2,3.7)
Lao People's Democratic Republic	277122 (254762,299243	10702 (9904.8,11504.6	621716 (570484,678042	10478.7 (9688,11293)	-2.1 (-4.4,0.4)
Malaysia	1328704 (1222289,1442379)	10828.3 (10076.2,11666.6)	3443338 (3171290,3729769)	11008.6 (10171.2,11849.4)	1.7 (-1,4.4)
Maldives	12689 (11699,13709)	10424.9 (9701.4,11220.8	52048 (47116,57976)	10202.7 (9432,11087)	-2.1 (-4.6,0.4)
Mauritius	96542 (88907,105229)	10712.2 (9939.1,11620.8)	189008 (175935,203155)	11411.6 (10649.1,12263.7)	6.5 (3.3,10)
Myanmar	2982783 (2758045,3235228)	10357.2 (9596.6,11185.4)	5373282 (4929525,5841826)	10173 (9393.1,11017.3)	-1.8 (-4.2,0.8)
Philippines	4286723 (3941958,4660864)	10549.8 (9776.7,11356.1)	10193697 (9395294,11071571)	10530.4 (9775.3,11372.9)	-0.2 (-0.9,0.4)
Sri Lanka	1386481 (1270757,1506959)	10274.6 (9504.3,11118.7)	2647885 (2443533,2855363)	10450.5 (9660.4,11299.2)	1.7 (-0.7,4.5)
Seychelles	6521 (6032,7044)	10613 (9840.2,11453.2	12837 (11856,13936)	10884.5 (10070.5,11753)	2.6 (-0.3,5.5)

Thailand	4699377 (4333121,5101891)	10448.1 (9717.1,11188.5)	10328746 (9637500,11140578)	10839.2 (10107.8,11724.9)	3.7 (1.2,6.6)
Timor-Leste	45151 (41110,49397)	9936.4 (9194.9,10715.2	104170 (96163,112715)	10410.8 (9601.8,11301.7	4.8 (2.3,7.3)
Viet Nam	4431264 (4056446,4826467)	9094.4 (8336.9,9858.1)	9699996 (8886323,10558442)	9152.1 (8434.8,9895.8)	0.6 (-1.8,3.2)
Oceania	306814 (282467,332777	7742.4 (7231.4,8304.2)	776661 (715870,846878	7950.7 (7408.8,8567.7)	2.7 (0.7,4.4)
American Samoa	2729 (2519,2958)	8831.3 (8197.4,9501.6)	4468 (4153,4802)	9256.7 (8599,9943)	4.8 (2.1,7.6)
Cook Islands	1150 (1062,1242)	8019.6 (7447.9,8644.5)	1841 (1712,1970)	8230 (7649.9,8830.4)	2.6 (0.2,5.2)
Micronesia(Federated States of)	5110 (4740,5505)	8313.3 (7738.6,8898.9)	7394 (6849,8003)	8744.1 (8145.4,9371.5)	5.2 (2.8,7.7)
Fiji	43084 (39626,46792)	8584.3 (7980.7,9231)	74318 (68583,80409)	8913.8 (8286.6,9600.9)	3.8 (1.2,7)
Guam	8188 (7532,8928)	7935.1 (7378.4,8544.5)	16160 (14994,17291)	8480.2 (7890.5,9106)	6.9 (4.2,9.6)
Kiribati	4029 (3716,4354)	8459.6 (7856.8,9093.5)	7836 (7239,8460)	8718.2 (8109.9,9329.4)	3.1 (0.5,5.6)
Marshall Islands	1905 (1760,2065)	8175.3 (7594.6,8766.6)	3728 (3432,4030)	8476 (7879.7,9042)	3.7 (1.3,6.2)
Nauru	544 (502,592)	8632.1 (8048,9264.7)	698 (644,757)	9105 (8473.2,9748.9)	5.5 (3.3,7.8)
Niue	186 (173,199)	8670.5 (8041.9,9334)	175 (162,188)	8957.5 (8282.6,9663)	3.3 (1.1,5.7)
Northern Mariana Islands	2867 (2626,3141)	8797.6 (8200.9,9441.4)	4746 (4387,5099)	9078.8 (8455.6,9714.1)	3.2 (0.7,5.8)
Palau	1027 (956,1114)	8544.2 (7955.6,9159.8)	1947 (1797,2108)	8900.4 (8266.8,9595.2)	4.2 (1.7,6.8)
Papua New Guinea	179603 (165528,195405	7388.6 (6896.2,7935.3)	538462 (494564,590048	7685.7 (7155,8282.9)	4 (1.4,6.2)
Samoa	8628 (8012,9299)	8335.3 (7760,8940.8)	13962 (12935,15079)	8626.9 (8017.8,9290.7)	3.5 (0.9,6)
Solomon Islands	14747 (13720,15956)	8066.7 (7526,8676.1)	37933 (34962,41296)	8231.6 (7603.5,8851.1)	2 (-0.9,4.9)
Tokelau	107 (99,114)	8149.5 (7578,8739.1)	121 (112,130)	8484 (7876.5,9158.2)	4.1 (1.7,6.9)
Tonga	5372 (4996,5776)	8442.8 (7847.4,9065.9)	7502 (6986,8081)	8763.8 (8180,9417.8)	3.8 (1.3,6.2)
Tuvalu	609 (566,656)	8324.3 (7745.8,8960.2)	948 (879,1026)	8641.4 (8027.9,9325)	3.8 (1.5,6.3)
Vanuatu	7356 (6741,7986)	8514.5 (7893.4,9206.9)	19551 (18111,21165)	8886.1 (8277.8,9532.4)	4.4 (1.9,7.2)
North Africa and Middle East	19739135 (18244354,21279746)	9119.7 (8490.9,9769.6)	49668756 (45835433,53616786)	9180 (8523.5,9830.1)	0.7 (-0.3,1.8)
Afghanistan	611982 (569081,656341)	8808.9 (8175.3,9452.3)	1440765 (1317910,1571513)	8934.9 (8262.8,9627.3)	1.4 (-0.7,3.5)
Algeria	1413887 (1302734,1527151)	8946 (8267.2,9615.9)	3553584 (3275804,3852065)	8807.2 (8150,9515.2)	-1.6 (-3.7,0.9)

Bahrain	31525 (28728,34958)	9152.1 (8487.5,9866.9)	130181 (119335,142831	9108.5 (8445.2,9765.8)	-0.5 (-2.7,1.9)
Egypt	3069526 (2834187,3319651)	8613.6 (8031.7,9206)	7320032 (6762507,7887829)	9167.8 (8515,9825.4)	6.4 (4.3,8.7)
Iran (Islamic Republic of)	3141831 (2920460,3371296)	9513.7 (8874,10180.4)	8145077 (7562399,8737333)	9474.8 (8837.1,10118.4)	-0.4 (-1.5,0.6)
Iraq	1026557 (949992,1108210)	9536.9 (8851.5,10239.3)	2977727 (2740400,3234895)	9464.2 (8764.4,10188.2)	-0.8 (-3.1,1.6)
Jordan	187654 (172497,203471	9249.5 (8606.8,9945.6)	889238 (817131,968041	8989.7 (8318.6,9729.3)	-2.8 (-5.4,-0.1)
Kuwait	106214 (96298,117759)	8982.8 (8321.1,9651.6)	393166 (356902,436552	8593.1 (7931.8,9269.4)	-4.3 (-6.9,-1.4)
Lebanon	211783 (195737,228352	8827.6 (8183.8,9494.6)	548008 (508010,589947	8988.2 (8309.1,9607.8)	1.8 (-0.4,4.2)
Libya	228746 (211473,246316	9024.4 (8360,9727.3)	610601 (560616,667943	9269.1 (8567.5,10032.2	2.7 (0.4,5)
Morocco	1521333 (1399644,1649317)	8496.6 (7864,9155.1)	3125307 (2880319,3372664)	8498.4 (7864,9135)	0 (-2.2,2.3)
Palestine	105072 (97202,113180)	9224.6 (8569.8,9850.2)	316240 (292025,342754	8942.6 (8314.5,9603.5)	-3.1 (-5.4,-0.4)
Oman	98358 (89680,108123)	8427.7 (7838.2,9068.7)	344284 (313617,379720	9024.5 (8385.7,9702.4)	7.1 (4.6,9.6)
Qatar	28926 (26054,32385)	9257.3 (8540.3,9961.5)	238389 (214245,267350	9111.2 (8448,9852.6)	-1.6 (-4.4,1.4)
Saudi Arabia	854299 (786274,926817)	9213.2 (8560.7,9892.3)	3067523 (2798321,3367524)	9245.5 (8568.2,9935.6)	0.4 (-2.2,2.8)
Sudan	1061226 (976894,1142517)	8869.2 (8238.7,9502)	2607644 (2388823,2834157)	9227.5 (8545.5,9928.6)	4 (1.6,6.4)
Syrian Arab Republic	651505 (602699,705033)	9137.9 (8502.5,9774.2)	1213830 (1120670,1309607)	8947.4 (8264.7,9632.4)	-2.1 (-5,0.7)
Tunisia	522181 (483654,562934)	8690.3 (8101.6,9339.4)	1144106 (1055790,1229704)	8690.2 (8056.9,9347.2)	0 (-2.3,2.6)
Turkey	4172206 (3844649,4504302)	9813.7 (9115.6,10517.9)	8863959 (8169195,9513046)	9503.7 (8759.6,10189.4)	-3.2 (-5.6,-0.6)
United Arab Emirates	123088 (111138,138046)	9906.1 (9179.7,10668.9)	940579 (846409,1053023)	9512.7 (8796.6,10259.4)	-4 (-6,-1.6)
Yemen	560437 (518019,609175)	8318 (7725.8,8913.9)	1752190 (1608715,1906736)	8511.9 (7887.1,9137.9)	2.3 (-0.1,4.5)
South Asia	72584182 (67087414,78502263)	9936.6 (9252.6,10645.7)	158803354 (147190732,171556959)	9565.3 (8903.7,10265.7)	-3.7 (-4.7,-2.8)
Bangladesh	5331909 (4950686,5772489)	8395.6 (7832.8,8968.1)	12882108 (11949483,13899210)	8555.4 (7963.4,9177.3)	1.9 (-0.5,4.5)

Bhutan	31715 (29384,34240)	9135.3 (8522.1,9749.9)	64186 (59913,69314)	9213.5 (8612.1,9876.6)	0.9 (-1.5,3.3)
India	60111283 (55460535,65038616)	10258.7 (9547.9,10986.1)	128031911 (118513994,138721278)	9710.5 (9024.2,10420.3)	-5.3 (-6.4,-4.4)
Nepal	1177468 (1101596,1257964)	10501.2 (9843.1,11204.3)	2762106 (2575557,3032129)	10887.7 (10177.4,11883.1)	3.7 (0.9,13)
Pakistan	5931806 (5491196,6396491)	8589.1 (7994.6,9202.4)	15063043 (13945283,16289223)	9164.6 (8522.8,9774.2)	6.7 (5.2,8.3)
Southern Sub-Saharan Africa	2945199 (2740170,3170275)	8946.4 (8370.4,9563.7)	5972951 (5547456,6415524)	9037.9 (8440.6,9647.1)	1 (-0.2,2.4)
Botswana	60835 (56449,65830)	8684.7 (8075.9,9285.6)	164894 (153184,177219	8867.3 (8244.1,9441.2)	2.1 (-0.6,5)
Lesotho	79823 (74328,85461)	8435.2 (7898.7,8992.7)	114258 (105784,123018	8824.6 (8201.2,9408.8)	4.6 (1.7,7.6)
Namibia	68175 (63117,73308)	8664.4 (8056.5,9233.5)	144692 (133912,155904	8510.1 (7908.4,9112.4)	-1.8 (-4.1,0.9)
South Africa	2257302 (2099020,2428551)	9062.8 (8465.3,9681)	4677573 (4347292,5025759)	9097.4 (8501.9,9729.1)	0.4 (-0.9,1.8)
Eswatini	33811 (31458,36377)	8783.2 (8183.6,9417.6)	66871 (61736,72023)	9047.6 (8414.1,9624.3)	3 (0.3,5.8)
Zimbabwe	445253 (413595,477795	8575.2 (7995.1,9192.8)	804662 (746942,870315	8832.9 (8243,9439.3)	3 (0.4,5.7)
Western Sub-Saharan Africa	9003658 (8381320,9663357)	8310.7 (7767.4,8851.6)	22472497 (20882526,24191820)	8324.3 (7782.3,8871)	0.2 (-0.9,1.2)
Benin	188015 (174766,201634	7343.4 (6857.5,7853.5)	552484 (512421,599062	7482.3 (7003.2,8021.9)	1.9 (-0.8,4.6)
Burkina Faso	365246 (337985,392297	7076.2 (6568.6,7570.5)	901049 (834507,973982	7181.8 (6699.9,7686.7)	1.5 (-1,4)
Cameroon	463067 (431742,496225)	8270.7 (7742.1,8827.3)	1594660 (1486358,1713045)	9043.6 (8503.4,9609.8)	9.3 (6.2,12.5)
Cabo Verde	17443 (16294,18677)	7283.3 (6802.8,7767.9)	38020 (35307,41075)	7366.7 (6831.1,7880.3)	1.1 (-1.3,3.4)
Chad	241571 (223146,258353	7195.1 (6688.4,7683.2)	603512 (558192,650782	7271 (6764.7,7787.6)	1.1 (-1.5,3.8)
CÃ´te d'Ivoire	462576 (427133,500797)	7562 (7049.4,8106.4)	1249475 (1157292,1360894)	7597.7 (7075.2,8139.2)	0.5 (-2.1,2.9)
Gambia	36537 (33712,39478)	7285.8 (6764.4,7776.6)	103566 (95771,112154)	7469.2 (6954.6,8002.9)	2.5 (-0.2,5.3)
Ghana	516871 (472923,562875)	5943.1 (5509.3,6423.8)	1422793 (1305227,1553890)	6033.8 (5584.9,6513.2)	1.5 (-0.7,4.2)
Guinea	275726 (256995,296333	7313.9 (6820.2,7803.8)	568301 (528813,612479	7498.3 (6998.8,7995.5)	2.5 (0.2,5.1)
Guinea-Bissau	40061 (37305,43253)	7641.8 (7095.7,8167.3)	85214 (78709,92411)	7643.8 (7104.1,8145.4)	0 (-2.4,2.5)
Liberia	107783 (99699,116096)	7548.2 (7002.1,8076.2)	244755 (226016,265719	7551.2 (7049.8,8085.2)	0 (-2.3,2.4)

Mali	358053 (333871,381898	7349.8 (6875.5,7816.7)	909702 (841914,979718	7419.9 (6929.5,7932.6)	1 (-1.5,3.4)
Mauritania	91156 (84420,98252)	7479.4 (6957.7,8019.8)	197970 (183860,213616	7285.5 (6794.6,7783.8)	-2.6 (-4.9,-0.3)
Niger	265038 (246140,284905	6880.2 (6411.5,7331.4)	790430 (731816,852136	6881.8 (6387.8,7357.1)	0 (-2.9,2.6)
Nigeria	4951044 (4609650,5305923)	9402.3 (8798.4,10034.7)	11730967 (10926433,12598672)	9488.9 (8881.1,10113.3)	0.9 (-0.2,2.1)
Sao Tome and Principe	5810 (5424,6228)	7967.4 (7434.1,8520.1)	12272 (11408,13236)	8194.6 (7639.2,8749.2)	2.9 (0,5.9)
Senegal	294300 (271578,318380	6986.3 (6472.3,7529.8)	679249 (627366,734863	6614 (6185.7,7067.8)	-5.3 (-7.6,-2.6)
Sierra Leone	191300 (177895,206334	7521.3 (7006.4,8070.4)	405349 (372950,439223	7610.6 (7071.5,8155.3)	1.2 (-1.1,3.7)
Togo	131761 (121749,142772	7346 (6804,7869)	382466 (353706,416128	7283.9 (6781.1,7815.9)	-0.8 (-3.4,1.5)
Eastern Sub-Saharan Africa	5981606 (5488507,6503182)	5677.2 (5266.2,6113.1)	15017846 (13741759,16422384)	5821.3 (5404.5,6293.6)	2.5 (1.7,3.3)
Burundi	187278 (170970,204259	5868.1 (5412.1,6352.8)	456223 (414259,502680	5921.6 (5472.5,6436.6)	0.9 (-1.4,3.5)
Comoros	15628 (14361,16936)	5870.7 (5455.7,6305.4)	35339 (32389,38509)	5851.8 (5407.4,6323.6)	-0.3 (-2.6,2.3)
Djibouti	13012 (11876,14272)	5545.1 (5142.1,5961.8)	55546 (50733,60942)	5776.9 (5342.9,6232.5)	4.2 (1.8,6.5)
Eritrea	99470 (90769,109284)	5529.9 (5117.9,5982.1)	244600 (224167,268056	5676.6 (5255.1,6117.6)	2.7 (0.2,4.8)
Ethiopia	1542523 (1422919,1673239)	5569.3 (5168.8,5985.1)	3762262 (3443544,4113603)	5649.3 (5230.3,6098.2)	1.4 (-0.1,3)
Kenya	700742 (642264,764896)	5761.7 (5343.2,6221.9)	1979251 (1807371,2167364)	5900.7 (5470,6369)	2.4 (1.8,3)
Madagascar	389060 (359027,423079)	5625.8 (5196.6,6061.5)	994473 (908917,1089154)	5685.3 (5246.2,6164.1)	1.1 (-1.3,3.5)
Malawi	326018 (298440,355273	5918.5 (5492,6409.4)	705439 (644778,770229	6124.8 (5658.2,6619.3)	3.5 (1,5.8)
Mozambique	471644 (433799,513878)	5918.7 (5479.6,6395.1)	1066862 (978804,1170332)	6162.6 (5705.7,6708.9)	4.1 (1.8,6.6)
Rwanda	234499 (214398,255853	5852.6 (5422.1,6320)	513932 (471486,561218	5696.5 (5277.2,6164.1)	-2.7 (-5,-0.2)
Somalia	224333 (205558,246035	5516.9 (5100.6,5954.8)	603690 (549230,663805	5565 (5152.6,6029.6)	0.9 (-1.3,3.1)
South Sudan	193641 (177480,211370	5618.8 (5175.1,6089.5)	323627 (296973,354259	5730.3 (5317.9,6182.2)	2 (-0.4,4.3)
United Republic of Tanzania	793891 (729869,860415)	5368.9 (4960.7,5794.8)	2186544 (2008467,2390011)	5861.5 (5421.9,6348.6)	9.2 (6.6,11.7)
Uganda	554191 (507911,603268)	6119.9 (5653.8,6571.6)	1414668 (1296989,1547642)	6081.4 (5638.5,6582.4)	-0.6 (-2.9,1.6)

Zambia	231401 (212298,251949	5513.6 (5099.7,5947.9)	662316 (603070,727722	5769.9 (5330.9,6256.6)	4.6 (2.2,7.2)
Central Sub-Saharan Africa	2633228 (2443845,2831336)	9222 (8598.7,9806.7)	6811951 (6363747,7313187)	9165.2 (8640.8,9709.7)	-0.6 (-2.5,1.6)
Angola	473841 (440001,510739)	9090.8 (8510.8,9718.4)	1482282 (1374318,1605870)	9018.9 (8383.2,9636)	-0.8 (-3.2,1.7)
Central African Republic	133406 (123251,143340	9207.4 (8552.5,9860.3)	276101 (255353,297770	9141.2 (8513.3,9769.2)	-0.7 (-3.3,1.8)
Congo	122987 (114132,132085	9370.1 (8763.1,9998)	331895 (307907,357776	9319.6 (8696.9,9931.8)	-0.5 (-3.1,1.7)
Democratic Republic of the Congo	1824071 (1691866,1955647)	9255.3 (8613.5,9831.1)	4529099 (4238150,4861233)	9196.8 (8707.1,9726.6)	-0.6 (-3.3,2.3)
Equatorial Guinea	20935 (19447,22604)	9087.3 (8498.5,9734.5)	75546 (69523,82103)	9342.3 (8719.9,9945.4)	2.8 (0.5,5.3)
Gabon	57987 (53943,62001)	9126 (8487.1,9779.8)	117028 (108707,125772	9238 (8645.4,9881)	1.2 (-1.6,3.7)

Table S2: Deaths due to chronic kidney disease from 1990 to 2021 and the percentage change in the age-standardised mortality rates (ASMRs) per 100,000, by location					
	1990		2021		Percentage change in the ASRs per
	No (95% UI)	ASRs per 100,000 (95%	No (95% UI)	ASRs per 100,000 (95%	
Global	552673 (513463,607915)	14.9 (13.6,16.4)	1527639 (1389377,1638914)	18.5 (16.7,19.9)	24.5 (10.2,33.5)
High-income North America	29949 (27171,31433)	8.3 (7.6,8.7)	143679 (124827,154757	20.6 (18.1,22)	147 (139.1,155.4)
Canada	2119	6.8 (6.1,7.2)	7789	9.5 (8.2,10.3)	40.1
Greenland	3 (3,4)	13.1 (11,15.3)	8 (6,10)	14.8 (10.7,18.6)	12.9 (-
United States of America	27826 (25245,29210)	8.5 (7.7,8.9)	135880 (118167,146606	21.9 (19.3,23.5)	158.3 (149.6,167.9)
Australasia	1851	8.5 (7.7,9)	5968	9.6 (8.2,10.5)	13.7 (6.1,20.4)
Australia	1580	8.7 (7.9,9.3)	5011	9.4 (8,10.3)	7.9 (0,15.4)
New Zealand	271 (249,288)	7.3 (6.6,7.7)	957 (835,1041)	10.7 (9.4,11.6)	47.1
High-income Asia Pacific	22233 (20303,23333)	12.6 (11.3,13.3)	63459 (50432,70953)	9.7 (8.1,10.7)	-22.8 (-28.7,-18.6)
Brunei Darussalam	32 (26,42)	37.5 (30.7,47.8)	86 (73,100)	36.3 (30.6,42.3)	-3.2 (-24.4,21)
Japan	17679 (16010,18557)	11.6 (10.4,12.3)	52717 (41038,59205)	9.5 (7.8,10.4)	-18.3 (-24.2,-14.1)
Singapore	264 (250,275)	13.7 (12.8,14.4)	833 (718,926)	10.2 (8.7,11.3)	-25.9 (-33.4,-18.5)
Republic of Korea	4257 (3775,4740)	18.6 (16.3,21.4)	9823 (7902,11473)	10.8 (8.7,12.7)	-41.8 (-53,-31.5)
Western Europe	48662 (44430,50877)	8.3 (7.5,8.7)	133481 (109637,148166	10.7 (8.9,11.8)	29 (18.4,37.6)
Andorra	5 (4,7)	12.1 (8.8,16.7)	17 (12,23)	9.3 (6.9,12.5)	-23.3 (-48.3,11.8)
Austria	786 (724,834)	6.6 (6.1,7)	3411	14.3 (11.9,15.6)	117.1
Belgium	1386	8.8 (8,9.4)	2816	9 (7.5,10)	2.1 (-7.4,10.6)

Cyprus	188 (156,238)	41.3 (34.1,51.5)	343 (284,403)	21.5 (18.1,25)	-47.8 (-58.9,-36.5)
Denmark	413 (379,437)	4.8 (4.5,5.1)	1699 (1457,1893)	12.1 (10.5,13.5)	150.4 (127.7,173.3)
Finland	214 (194,227)	3 (2.7,3.2)	818 (657,927)	4.9 (4,5.5)	61.4 (44.1,77)
France	6888 (6157,7355)	7.7 (6.9,8.3)	15133 (12606,16843)	7.3 (6.2,8.1)	-5.4 (-13.7,2.3)
Germany	11051 (10128,11739)	8.3 (7.6,8.8)	39563 (31943,45262)	15.4 (12.6,17.4)	85 (64.7,105.6)
Greece	2985	21.3 (19.4,22.7)	7053	21.3 (18.7,23)	0.1 (-6.4,6)
Iceland	11 (10,12)	3.6 (3.2,3.9)	41 (33,46)	5.8 (4.7,6.5)	61.2
Ireland	311 (288,330)	8.2 (7.5,8.7)	703 (587,792)	8.2 (6.9,9.2)	-0.2 (-
Israel	970 (893,1037)	22.2 (20.2,23.8)	2790 (2314,3075)	19.7 (16.5,21.7)	-11.2 (-19.6,-4.7)
Italy	7528 (6774,7922)	8.8 (7.8,9.2)	19086 (15314,21515)	9.3 (7.6,10.5)	6 (-3.6,15)
Luxembourg	52 (49,55)	10.3 (9.5,10.9)	158 (130,184)	12.6 (10.5,14.5)	22.6 (6.4,40.3)
Malta	46 (42,49)	12.1 (10.9,12.9)	141 (116,160)	12.5 (10.4,14.1)	3.1 (-9,15.6)
Monaco	5 (4,6)	6.1 (4.6,7.4)	12 (9,14)	9.6 (7.6,11.4)	58.1 (24.1,109.6)
Netherlands	1325	6.6 (5.9,7.1)	4435	10.9 (9.2,12.1)	63.8
Norway	268 (241,282)	3.5 (3.2,3.7)	805 (673,878)	6.5 (5.5,7)	83.5
Portugal	1597	13 (12,13.8)	4736	14.5 (12.3,16.2)	11.3 (-1.6,23.1)
San Marino	2 (2,2)	5.5 (4.5,6.6)	4 (3,6)	3.9 (2.6,5.3)	-28.9 (-50.9,-2.7)
Spain	6907 (6235,7340)	13.3 (11.9,14.1)	14802 (11854,16621)	10.5 (8.6,11.8)	-21.2 (-29.2,-13.8)
Sweden	636 (578,678)	3.8 (3.5,4.1)	2518 (2087,2834)	8.9 (7.4,9.9)	131.8 (106.5,154.6)
Switzerland	808 (722,860)	7.1 (6.4,7.6)	2599	10.2 (8.1,11.7)	42.8
United Kingdom	4239 (3908,4410)	4.6 (4.2,4.8)	9679 (8207,10541)	6.2 (5.4,6.8)	36 (28.4,42.2)
Southern Latin America	11256 (10698,11760)	26 (24.6,27.2)	21583 (19510,22986)	23.8 (21.6,25.4)	-8.3 (-14.1,-3)
Argentina	9185 (8697,9648)	30.4 (28.6,31.9)	15213 (13851,16266)	26.3 (24,28.1)	-13.3 (-19,-7.6)
Chile	1471	16 (15,16.9)	5202	19.8 (17.5,21.3)	23.8
Uruguay	598 (561,628)	15.5 (14.5,16.2)	1167	17.9 (16.1,19.4)	15.6 (6.1,25.2)
Eastern Europe	9389 (9183,9574)	3.6 (3.5,3.7)	17821 (16126,19853)	5.2 (4.7,5.8)	45.9 (33.3,59.9)
Belarus	100 (93,106)	0.8 (0.8,0.9)	360 (296,427)	2.4 (1.9,2.8)	182.4 (130,239.1)
Estonia	120 (112,127)	6.4 (6,6.8)	527 (437,605)	17 (14.2,19.7)	166.6 (125.1,206.6)
Latvia	95 (89,101)	2.9 (2.7,3.1)	321 (265,392)	7.7 (6.4,9.5)	166.7 (118.1,224.5)
Lithuania	104 (97,109)	2.5 (2.3,2.6)	332 (281,400)	5.8 (4.9,7.1)	136 (98,182.8)
Republic of Moldova	83 (79,87)	2 (1.9,2.1)	206 (179,242)	3.6 (3.2,4.2)	78.7 (56.2,109.6)
Russian Federation	8804 (8612,8979)	5.1 (5,5.2)	14471 (13013,16149)	6.2 (5.6,6.9)	20.2 (9.3,32.5)
Ukraine	84 (80,88)	0.1 (0.1,0.1)	1605 (1204,2049)	2.4 (1.8,3.1)	1714.7 (1257.3,2220.9
Central Europe	14335 (13732,14892)	10.5 (10,10.9)	21635 (19445,23977)	9.4 (8.4,10.5)	-10.4 (-18.3,-1.7)

Albania	234 (196,277)	12 (10,14.3)	424 (322,541)	10.6 (8.1,13.4)	-11.6 (-34.7,19.9)
Bosnia and Herzegovina	361 (307,422)	9.8 (8.3,11.4)	686 (512,869)	11 (8.3,13.9)	12.5 (-17.4,45.7)
Bulgaria	729 (668,785)	7.6 (7,8.2)	2428 (2049,2872)	17.4 (14.6,20.8)	127.8 (87.6,171.4)
Croatia	502 (464,540)	9.5 (8.8,10.2)	1337	13.5 (11.6,15.7)	42.2
Czechia	1096 (999,1200)	8.2 (7.5,8.9)	1562 (1326,1820)	6.8 (5.8,8)	-16.8 (-30.1,-1.3)
Hungary	842 (795,888)	6.3 (5.9,6.6)	2173	10.1 (8.7,11.6)	61.8
Montenegro	76 (61,93)	12.9 (10.4,15.8)	165 (124,208)	19.1 (14,23.9)	47.8 (-
North Macedonia	173 (145,208)	10.1 (8.5,12.2)	370 (282,484)	13.6 (10.7,17.3)	34.8 (0.7,78.6)
Poland	5309	12.8 (12.3,13.2)	4981	6.6 (5.8,7.6)	-48.5 (-53.7,-
Romania	2551 (2408,2682)	10.4 (9.8,10.9)	3325 (2869,3831)	8.6 (7.4,10)	-17.3 (-29.8,-3.2)
Serbia	1464	16.5 (13.6,20.8)	2713	15.9 (12.8,19.1)	-3.7 (-
Slovakia	617 (530,708)	10.6 (9.1,12.2)	831 (677,1002)	8.9 (7.3,10.7)	-16 (-32.9,3)
Slovenia	152 (142,163)	6.4 (5.9,6.9)	327 (261,415)	6.1 (4.9,7.8)	-4.3 (-
Central Asia	2536 (2250,2933)	5 (4.4,5.9)	9397 (8278,10515)	12.1 (10.7,13.5)	141.9 (101.3,186.6)
Armenia	27 (22,31)	1 (0.8,1.1)	432 (354,523)	10.3 (8.4,12.4)	953.1 (706,1362.9)
Azerbaijan	357 (277,452)	6.6 (5.1,8.3)	992 (717,1295)	10.4 (7.6,13.4)	57.8 (14.7,127.4)
Georgia	197 (161,247)	3.3 (2.7,4.1)	713 (574,898)	12.3 (9.9,15.5)	272.5 (194,355.8)
Kazakhstan	737 (644,832)	5.3 (4.6,6)	1982	12.2 (10.1,14.4)	129.1
Kyrgyzstan	159 (141,180)	4.6 (4,5.2)	498 (415,596)	10 (8.3,11.9)	118.4 (74.7,169.5)
Mongolia	173 (139,213)	15.4 (12.4,18.9)	296 (236,365)	13.6 (10.8,16.8)	-11.5 (-34.5,15.1)
Tajikistan	59 (48,83)	1.8 (1.4,2.6)	152 (106,220)	2.4 (1.7,3.3)	34.2 (-7.7,93.9)
Turkmenistan	209 (191,228)	8.9 (8.1,9.8)	744 (574,947)	18 (14,22.7)	101.4 (53.2,162.9)
Uzbekistan	617 (441,920)	5 (3.4,7.9)	3589	14.2 (12.1,16.7)	182.1
Central Latin America	22182 (21513,22820)	27.9 (26.8,28.8)	104444 (94171,116461)	42.4 (38.3,47)	51.6 (37.5,66.7)
Colombia	3340 (3177,3471)	19.5 (18.4,20.4)	8646 (7184,10116)	15.5 (12.9,18.1)	-20.7 (-33,-8)
Costa Rica	264 (247,279)	15.3 (14.2,16.2)	1605 (1391,1794)	29 (25.2,32.3)	89.7 (69.5,112.5)
El Salvador	797 (702,1141)	25.3 (22.1,36.3)	4441 (3314,5504)	69.8 (52.2,86.8)	176.3 (60.6,259.3)
Guatemala	1108	33.7 (32,35.3)	5448	50.7 (42.3,59.3)	50.4 (23.7,78)
Honduras	259 (215,328)	12.3 (10.1,16)	1439	24.6 (18.8,31.7)	99.5 (27,172.3)
Mexico	14133 (13701,14565)	36.9 (35.4,38.4)	67044 (58757,76983)	54.3 (47.8,61.9)	47 (29.7,64.9)
Nicaragua	492 (444,663)	29.4 (26.2,40.1)	2686	55.3 (44.7,66.5)	88.4 (29.6,136)
Panama	201 (188,215)	13.5 (12.6,14.5)	1381 (1090,1653)	30.7 (24.3,36.8)	127.6 (80.4,176.6)
Venezuela （Bolivarian Republic of)	1587 (1501,1651)	16.3 (15.2,17.1)	11753 (8833,14984)	40.5 (30.4,51.3)	148.2 (90.2,213.8)
Andean Latin America	5795 (5288,6456)	28.5 (25.9,31.9)	21619 (17903,25800)	37.7 (31.3,44.9)	32 (7.7,63.3)

Bolivia (Plurinational State of)	1325 (1115,1675)	43 (36.2,53.9)	4731 (3675,6140)	58.2 (45.7,75.3)	35.5 (-0.3,86.9)
Ecuador	1227 (1150,1287)	23.7 (22.1,25)	6502 (4898,8822)	42.1 (32.2,56)	77.5 (35.9,133.3)
Peru	3242 (2796,3748)	27 (23.3,31.4)	10386 (7395,13295)	30.9 (22.1,39.7)	14.6 (-18.4,51.8)
Caribbean	4754 (4399,5437)	18.7 (17.3,21.3)	13888 (12086,16193)	25.8 (22.4,30.1)	37.6 (18.9,57.5)
Antigua and Barbuda	17 (16,18)	30.1 (27.9,32.2)	46 (42,49)	47.1 (43,50.5)	56.3
Barbados	60 (57,64)	20.4 (19.1,21.4)	148 (117,182)	29.3 (23.3,36)	43.8
Belize	24 (23,26)	24.4 (22.8,26)	134 (117,151)	45.5 (39.5,51.1)	86.3 (61.3,110.6)
Bermuda	9 (9,10)	15.5 (14.5,16.5)	23 (19,27)	15.7 (13.2,19)	1 (-15,22)
Bahamas	37 (34,40)	23.1 (21.3,25.1)	144 (117,178)	37.5 (30.7,46.2)	62.4 (30.1,104.3)
Cuba	831 (789,866)	8.3 (7.9,8.7)	3209 (2758,3638)	16.1 (13.9,18.3)	94.3 (69.1,121.4)
Dominica	19 (16,21)	32.4 (28.2,36.3)	40 (32,49)	50.3 (40.5,61.8)	55.4
Dominican Republic	621 (533,750)	16.7 (14.3,20.3)	2392	24 (16.5,30.7)	43.9 (-5.6,90.9)
Grenada	27 (24,29)	35.7 (32.5,39.2)	62 (54,69)	58.3 (51,64.9)	63.2 (40,87.3)
Guyana	117 (106,128)	30.5 (27.7,33.4)	349 (269,446)	56.5 (43.8,71.2)	85.3 (39.7,133.1)
Haiti	962 (707,1555)	28.7 (20.5,48.9)	2177	30.9 (17.2,64.8)	7.6 (-31.2,52.4)
Jamaica	434 (408,457)	23.3 (21.9,24.6)	935 (707,1205)	29.2 (22,37.5)	24.9 (-5.1,62.3)
Puerto Rico	1091	31.8 (29.9,33.4)	2537	32.1 (26.6,37.9)	1.2 (-15.9,18.5)
Saint Kitts and Nevis	16 (15,17)	43.6 (40.5,46.8)	34 (28,40)	57.6 (48.5,65.8)	32.1 (9.6,52.8)
Saint Lucia	30 (29,32)	37.3 (35.3,39.2)	93 (77,111)	40.3 (33.5,47.9)	8 (-11.2,27.6)
Saint Vincent and the Grenadines	19 (17,20)	26.9 (24.9,28.7)	54 (48,61)	41.1 (36.2,46.4)	52.7 (32.3,74.3)
Suriname	71 (61,81)	27.7 (24.2,31.8)	253 (189,329)	41.4 (31,53.6)	49.6
Trinidad and Tobago	192 (184,201)	24.7 (23.5,25.8)	755 (564,967)	40.2 (30.1,51.3)	62.6 (22,108.7)
United States Virgin Islands	15 (12,19)	19.9 (16.6,24)	33 (23,43)	20.5 (14.7,26.6)	2.9 (-32.5,44)
Tropical Latin America	15524 (14832,16076)	17.9 (16.9,18.7)	46925 (42508,49438)	18.8 (17,19.9)	5 (-0.5,9.1)
Brazil	15168 (14505,15699)	18 (16.9,18.8)	45537 (41271,47987)	18.7 (16.9,19.7)	4 (-1.2,8.1)
Paraguay	355 (301,418)	16.1 (13.6,19.1)	1389	24.9 (18.7,31.5)	54.4
East Asia	107701 (94477,126024)	14.4 (12.7,17)	217342 (178047,259057	11.1 (9.2,13.2)	-22.5 (-39.2,-5.5)
China	102726 (89745,120908)	14.2 (12.4,16.8)	204230 (164736,246372	10.8 (8.8,13)	-23.4 (-41,-5.7)
Democratic People's Republic of Korea	2021 (1438,2685)	14 (10.1,19.3)	4298 (3284,5596)	14 (10.7,18.3)	-0.5 (-29.5,40)
Taiwan (Province of China)	2954 (2802,3083)	24.1 (22.5,25.4)	8814 (7593,9771)	20 (17.4,22.1)	-17.1 (-24.6,-9.8)
Southeast Asia	57881 (52199,66490)	22.9 (20.6,26.7)	170033 (148864,190537	28.5 (24.8,31.9)	24.6 (2.4,43.3)
Cambodia	1050 (857,1277)	21.2 (17.5,25.7)	2681	23.5 (17.8,30.2)	10.7 (-25,54.2)
Indonesia	18490 (15846,22280)	17.3 (14.9,21.8)	51165 (41553,63408)	23 (18.8,29)	32.6 (2.9,60.8)
Lao People's Democratic Republic	917 (677,1191)	43.9 (33.2,56.9)	1775 (1242,2408)	40.9 (29.1,54.7)	-6.9 (-34.5,27.7)
Malaysia	2075	22.6 (19.4,25.9)	7575	29.2 (24.4,33)	29.2 (0.5,52.4)

Maldives	40 (34,51)	48.4 (39.7,69.3)	88 (71,105)	28.9 (23.4,34.5)	-40.3 (-57.8,-21.8)
Mauritius	302 (288,315)	44.3 (42,46.4)	1414	80.1 (74.1,84.6)	80.8
Myanmar	6501 (4977,8336)	26.6 (20.6,34.4)	11244 (9001,14131)	24.9 (20.3,31.1)	-6.1 (-33.4,31.9)
Philippines	8626 (7839,9922)	31.7 (28.9,35.6)	32883 (27438,38113)	41.7 (35,48.1)	31.5 (8.5,54)
Sri Lanka	2614 (2219,3080)	27.7 (23.3,32.8)	5243 (3567,7155)	21.2 (14.6,28.5)	-23.3 (-50.8,14.6)
Seychelles	17 (15,19)	29.3 (25.5,33.4)	50 (40,58)	47.2 (37.9,55.4)	61.2 (26.8,101.5)
Thailand	7941 (6624,9995)	24.3 (20.2,30.6)	31734 (24197,39501)	29.9 (22.8,37.1)	23.2 (-13.4,63.9)
Timor-Leste	76 (57,101)	25.6 (19.7,35.6)	238 (172,335)	29.8 (21.8,41.4)	16 (-19,61.6)
Viet Nam	9150 (6657,11695)	23.5 (17.1,30.1)	23706 (16677,29759)	27 (19.1,33.4)	15.2 (-18.9,51)
Oceania	489 (342,661)	17.2 (12.8,23.1)	1546	21.6 (18,26.4)	25.2 (-
American Samoa	7 (5,10)	33.5 (24.6,48.1)	31 (24,39)	73.8 (57.4,91.7)	120.1
Cook Islands	2 (2,3)	17.5 (13.8,23.2)	5 (4,7)	22 (16.6,27.2)	25.8 (-
Micronesia(Federated States of)	16 (11,25)	33.5 (22.8,54.5)	38 (27,53)	57.6 (42.1,80.9)	71.7 (4.1,175.3)
Fiji	95 (67,136)	27.7 (19.6,40.5)	314 (231,410)	48 (36.4,61.9)	73.1 (-
Guam	14 (12,17)	22.9 (20,28.3)	51 (42,58)	24 (20.4,27.2)	4.9 (-19.2,26.4)
Kiribati	11 (9,13)	30 (24.2,36.8)	29 (18,42)	43.1 (28,62.3)	43.9 (-13.2,125.4)
Marshall Islands	5 (3,9)	32.5 (20.2,58.7)	17 (6,49)	54.8 (17.4,160.8)	68.4 (-25.8,175.1)
Nauru	2 (1,3)	41.1 (26.1,68.8)	3 (2,5)	63 (42.9,105.4)	53.3 (1.6,138)
Niue	1 (1,1)	29.9 (23.1,40.3)	1 (1,2)	56.1 (33.1,86.2)	87.8
Northern Mariana Islands	6 (5,8)	37.3 (30.1,47.4)	25 (20,30)	57.6 (47.3,68.4)	54.7 (15.4,102)
Palau	3 (2,4)	29.5 (21.1,39.3)	9 (6,12)	50.2 (36.2,66.3)	70 (5.7,162)
Papua New Guinea	220 (145,312)	11.3 (7.9,15.9)	728 (561,945)	13.6 (10.4,19)	20.8 (-
Samoa	24 (17,33)	31 (22.2,41)	58 (44,75)	43.5 (33,55.5)	40.2 (-4,111.8)
Solomon Islands	31 (13,50)	22.1 (11.6,34.6)	90 (69,116)	25 (20.1,31.5)	13.1 (-
Tokelau	0 (0,1)	24.6 (17.1,42.5)	1 (0,1)	37.3 (27,54.7)	51.7
Tonga	7 (5,10)	13.4 (9.4,18.6)	16 (11,21)	20.6 (14.3,27)	53.6
Tuvalu	2 (1,3)	27.8 (20.8,43.6)	4 (3,6)	40.7 (29.7,58.9)	46.5 (-
Vanuatu	13 (8,22)	22 (14.3,38.7)	55 (40,85)	34.3 (24.7,52.6)	55.9
North Africa and Middle East	45472 (37703,66491)	31.2 (25.5,47)	144687 (126153,162699	37.7 (32.7,42.4)	20.8 (-23.7,50.5)
Afghanistan	3009	47.3 (34.5,75.9)	4893	53.9	13.9 (-
Algeria	2344 (1667,4128)	27.1 (19,47.7)	9785 (7909,12092)	37.3 (30.6,45.5)	37.6 (-25.2,107.8)
Bahrain	48 (36,63)	41.7 (31.3,55.7)	259 (207,316)	52.5 (42.1,63.4)	25.9 (-
Egypt	10610 (8478,16104)	51.8 (40.3,82.6)	33311 (27183,40816)	71.7 (59.7,86.6)	38.6 (-16.6,85.7)
Iran (Islamic Republic of)	3718 (2816,5017)	17.8 (13,24.7)	13627 (11262,15175)	20.2 (16.5,22.6)	13.6 (-26.4,43.6)
Iraq	2948 (2325,3775)	36.7 (28.7,47.6)	8279 (5635,10644)	43.4 (30.5,54.8)	18.3 (-29.7,66.6)
Jordan	360 (293,470)	31.6 (25.6,41.5)	1876	33.1 (26.2,41.8)	4.8 (-29.2,41)
Kuwait	160 (144,178)	30.7 (26.6,34.3)	374 (276,459)	16 (11.7,19.7)	-47.9 (-59.7,-35.3)
Lebanon	592 (455,790)	32.3 (24.7,42.7)	2102	31.9 (26.3,38.5)	-1.2 (-

Libya	503 (374,704)	28.1 (20.5,40.1)	2127 (1413,2796)	46.9 (31.3,61.1)	67.1 (-18.3,163.9)
Morocco	3070 (2131,5653)	23.1 (15.7,45.6)	11890 (8911,15306)	40 (30.3,51.3)	73 (-2.2,152.9)
Palestine	348 (260,460)	45.4 (34.2,61.6)	838 (711,1010)	43.2 (36.7,51.9)	-4.8 (-
Oman	140 (102,207)	22.9 (16.6,33.8)	616 (490,771)	41.7 (33,50.4)	82.1 (13.1,164.9)
Qatar	28 (16,43)	40.7 (22.7,66)	198 (153,254)	39.9 (32.4,49.2)	-2 (-40,73.3)
Saudi Arabia	2408 (1798,3342)	45.1 (33.5,64.1)	12226 (9096,15498)	79.3 (59.8,95.7)	75.8 (-4,151.8)
Sudan	2043	22.7 (15.9,43.5)	5602	32.1 (24.3,43.8)	41 (-
Syrian Arab Republic	1951	39.1 (30.4,52.8)	4836	47.7 (33.6,62.8)	22 (-32.2,80.6)
Tunisia	926 (706,1392)	22.2 (17,33.3)	3765	32.1 (23.1,43.9)	44.6 (-
Turkey	9286 (7515,12586)	30.6 (24.5,42.5)	24805 (19764,30524)	29.5 (23.3,36.4)	-3.5 (-35.5,26.8)
United Arab Emirates	83 (57,119)	23.1 (16,32.6)	672 (427,914)	37.5 (20.9,49.2)	62.8 (-11.2,133.6)
Yemen	871 (580,1629)	19.1 (12.7,38.9)	2471	20.6 (14.2,34.6)	7.8 (-26.8,59.5)
South Asia	79714 (68086,89404)	14 (12.1,15.9)	226043 (192546,264155	16.5 (14,19.3)	17.7 (-7.6,41.4)
Bangladesh	9553 (6572,11510)	14.9 (12.8,18.3)	15175 (11740,20173)	12.3 (9.5,16.2)	-17.6 (-36.9,6.2)
Bhutan	42 (30,58)	17 (12.4,24.2)	120 (81,165)	20.9 (14,28.5)	22.8 (-
India	58475 (50679,67536)	13.1 (11.2,15.2)	175637 (146072,208715	15.8 (13.1,18.7)	21 (-10.1,47.6)
Nepal	1249 (960,1663)	13.5 (10.4,18.1)	4048	19.2 (14.1,25.1)	42.5 (-
Pakistan	10395 (8266,13167)	18.8 (14.9,23.7)	31063 (24330,39844)	26.6 (21.4,33.9)	41.8 (7.9,85.4)
Southern Sub-Saharan Africa	5376 (4674,6619)	20.9 (18,26.2)	17365 (15612,19485)	34.4 (31.1,38.3)	65 (26.9,89.5)
Botswana	109 (79,157)	23.1 (17,32.1)	348 (270,479)	28.5 (23,39)	23.3 (-9.5,70.9)
Lesotho	153 (119,202)	19.9 (15.3,26.5)	445 (311,595)	46.2 (32.8,59.4)	132.6 (58.1,214.1)
Namibia	119 (92,169)	21.8 (16.7,31)	313 (231,422)	26.7 (20.3,35.2)	22.3 (-8.7,61.9)
South Africa	4020 (3508,4993)	19.7 (16.8,25.1)	13502 (12124,14916)	33.2 (29.6,36.6)	68.3 (27.7,94.4)
Eswatini	100 (79,125)	38.7 (30.5,48.3)	274 (174,383)	53.5 (36.5,71.6)	38.4 (-3.2,91.9)
Zimbabwe	874 (693,1148)	26 (20.5,34.2)	2483 (1893,3317)	41.4 (31.8,53.6)	59.3 (13.3,110.9)
Western Sub-Saharan Africa	28294 (24259,32687)	33.6 (29,39.2)	64796 (53512,76124)	36.4 (31.1,42.8)	8.2 (-9.3,24.9)
Benin	782 (673,912)	37.3 (32.3,44.7)	1940	39.4 (32.5,48.4)	5.6 (-16.1,31.4)
Burkina Faso	1555	38.3 (31.8,45.8)	3812	43.7 (35.3,53)	13.9 (-
Cameroon	2151	52 (40,67.1)	5928	51.2 (37.1,71.1)	-1.6 (-24,32.5)
Cabo Verde	38 (32,44)	15.4 (12.7,17.9)	124 (84,155)	28.9 (19.4,36)	88 (26.6,142.9)
Chad	751 (593,1013)	26.4 (20.5,36.9)	1811	31.6 (23.4,45.6)	19.7 (-6.2,53.1)
CÃ´te d'Ivoire	1455	38.9 (32,47.7)	4060	40.5 (33,51.2)	4.1 (-17.2,32.9)
Gambia	115 (90,143)	33.7 (26.8,41.3)	403 (305,513)	43.2 (33.2,54.7)	28.1 (-9.7,72.7)
Ghana	1745	29.5 (22.8,41.8)	7076	47.7 (37.9,59.3)	61.5 (-
Guinea	1055 (850,1342)	32.3 (25.5,41.8)	1874	34.5 (25.9,47.8)	6.8 (-19.3,41.1)
Guinea-Bissau	201 (157,246)	50.1 (40.3,60.7)	316 (246,404)	46.4 (37,58.9)	-7.5 (-
Liberia	533 (427,673)	46.4 (38,57.8)	954 (706,1263)	48.4 (36.3,63.3)	4.3 (-26.5,42.5)
Mali	1405	38.3 (31.3,47.7)	3001	37.4 (30.3,48.1)	-2.3 (-22,24.5)
Mauritania	394 (305,502)	42.6 (33.2,54.3)	774 (532,1095)	40.4 (27.5,56.5)	-5.2 (-
Niger	874 (706,1119)	29.5 (23.9,38.7)	2007	27 (19.9,40.1)	-8.6 (-

Nigeria	12895 (10282,15658)	31 (25.1,37.6)	25429 (19318,32599)	31.3 (25,38.1)	1 (-22,23.9)
Sao Tome and Principe	34 (28,38)	53.7 (45.3,60.8)	69 (50,85)	72.4 (51.4,86.9)	34.8 (-5,65.3)
Senegal	1300	40.6 (34.1,50.3)	3035	43.5 (33.6,59.2)	7.2 (-19.2,43.4)
Sierra Leone	628 (495,784)	30.1 (23.8,37.2)	1048 (802,1392)	29 (22.6,37.5)	-3.7 (-25,23)
Togo	381 (314,467)	31 (26.1,38)	1136 (854,1520)	34.5 (26.7,45.2)	11.5 (-
Eastern Sub-Saharan Africa	30615 (26919,35172)	42.4 (37.1,50.2)	60918 (53817,69935)	40.1 (35.6,46)	-5.5 (-18.6,8.4)
Burundi	909 (711,1148)	39.5 (31,50.6)	1515 (1109,2172)	35 (25.8,49.9)	-11.4 (-34.1,18.6)
Comoros	70 (54,88)	39 (30.8,48)	182 (132,235)	42.3 (31,54.4)	8.4 (-20.2,47.2)
Djibouti	35 (26,47)	29.3 (22.4,37.7)	211 (152,285)	41.8 (31.4,54.7)	42.5 (-3,99.6)
Eritrea	358 (267,498)	32.6 (24.7,45.5)	902 (610,1462)	38.1 (26.6,60.6)	16.7 (-8.6,54.5)
Ethiopia	12912 (10097,14949)	69 (57,79.6)	16918 (14059,20068)	42.4 (34.8,50.2)	-38.6 (-50.1,-25.5)
Kenya	1950	25.2 (19.6,40.8)	7330	37.6 (29.8,49.5)	49.1
Madagascar	1300	26.7 (21,38.2)	2660	27 (19.7,35.4)	1 (-28,34.3)
Malawi	1438	38.7 (31.2,49)	3141	45.4 (37.8,54.5)	17.5 (-
Mozambique	1433	26 (20.8,37.4)	3627	35.7 (26.9,48.8)	37 (-5.2,85.7)
Rwanda	1301	47.9 (39.1,57)	2068	38.7 (28,51.2)	-19.3 (-
Somalia	1051 (755,1454)	45.7 (33.4,63.2)	2791	47.9 (33.1,68.3)	4.9 (-19.6,35.3)
South Sudan	1009 (753,1391)	40.9 (31,56.2)	1949	55 (39.9,71.2)	34.3 (-
United Republic of Tanzania	3665 (3049,4635)	36.1 (30.3,46.2)	8860 (7121,11155)	37.7 (30.1,46.9)	4.4 (-22.1,38.5)
Uganda	1868	31 (23.5,40.7)	5279	38.8 (30.8,50.8)	25.3 (-8,75)
Zambia	1293	46.3 (39.5,54.3)	3432	52.2 (39.2,69.8)	12.8 (-
Central Sub-Saharan Africa	8667 (7200,10419)	42.7 (35.5,51)	21011 (16114,27467)	43.7 (33.3,56.3)	2.4 (-22.2,33.4)
Angola	1417	37.6 (29.3,46.9)	4073	39.2 (27.9,50.3)	4.1 (-24.4,40.1)
Central African Republic	507 (420,615)	47 (38.6,56.4)	963 (698,1352)	47.4 (35.4,63.9)	0.9 (-22.7,29.8)
Congo	531 (406,637)	55.8 (41,67.2)	1252 (847,1626)	54.8 (35.6,69)	-1.9 (-
Democratic Republic of the Congo	5868 (4671,7377)	42.3 (34.5,52)	13845 (9966,18759)	42.9 (31.4,57.7)	1.7 (-28.2,42.4)
Equatorial Guinea	79 (62,98)	43.8 (35,53.4)	255 (156,372)	54.7 (31.3,76.5)	25 (-27.8,84.7)
Gabon	265 (212,318)	51.1 (40.7,61.6)	621 (324,822)	71.5 (35.5,93)	40 (-15.4,87)

Table S3： DALYs due to chronic kidney disease from 1990 to 2021 and the percentage change in the age-standardised rates (ASRs) per 100,000, by location					
	1990		2021		Percentage change in the ASRs per
	No (95% UI)	ASRs per 100,000 (95%	No (95% UI)	ASRs per 100,000 (95%	
Global	20739895 (18843684,22588533)	479.8 (439.2,523.8)	44453684 (40840762,48508462)	529.6 (486.2,577.4)	10.4 (0.8,17.8)

High-income North America	895097 (798640,986360)	266.6 (238.3,293.2)	3120983 (2864207,3351576)	508.8 (467,545.4)	90.8 (82.4,100)
Canada	55398 (49172,61889)	178.4 (158.9,198.9)	150955 (134749,165748	224.7 (200.2,246)	26 (17.7,35.5)
Greenland	117 (102,135)	335.7 (294.9,381.6)	216 (178,257)	352.2 (285.9,418.3)	4.9 (-14.1,26)
United States of America	839563 (749127,924830)	276.1 (246.7,303.3)	2969764 (2728422,3188334)	543.3 (499.5,582.6)	96.8 (87.8,106.5)
Australasia	46405 (42217,50551)	211.1 (192.4,229.6)	113693 (101514,125301	216.6 (193.2,239.2)	2.6 (-2.5,7.1)
Australia	38617 (35222,42200)	211.2 (193.1,230.1)	93074 (82639,102916)	207.1 (183.5,229)	-1.9 (-7.3,2.8)
New Zealand	7788 (7047,8501)	211.3 (190.8,230.6)	20618 (18809,22453)	263.6 (241.2,286.6)	24.7 (17.4,32)
High-income Asia Pacific	599581 (550356,643830)	315.7 (289,339.2)	1146613 (990343,1268312)	235.6 (206,259.6)	-25.4 (-29.1,-22.3)
Brunei Darussalam	1038 (881,1248)	843.6 (710.9,1036.2)	2607 (2282,2940)	776.6 (676.5,884.2)	-7.9 (-25.2,11.5)
Japan	460213 (416011,499612)	290.4 (262.5,315)	923022 (792680,1028099)	239.9 (209.7,266.1)	-17.4 (-20.6,-14.7)
Singapore	8136 (7624,8708)	355.8 (333.3,379.5)	20705 (18435,23087)	251.9 (223.9,280.3)	-29.2 (-34.7,-23.6)
Republic of Korea	130194 (117908,142679	429.6 (388.7,470.4)	200278 (171976,226706	225.6 (193.7,255.8)	-47.5 (-54,-40.6)
Western Europe	1267566 (1125408,1409086)	234.1 (206.8,260.1)	2361342 (2067098,2638163)	241.7 (209.8,271.1)	3.2 (-1.7,8.5)
Andorra	148 (115,187)	286.9 (224.8,360.9)	357 (285,435)	229.2 (181.7,279.9)	-20.1 (-39.3,2.4)
Austria	23090 (20228,25966)	213.4 (187,238.5)	56356 (49530,62593)	288.2 (253.5,323.9)	35.1 (25.2,44.5)
Belgium	36155 (31529,40640)	248.1 (215.3,279.2)	55840 (47926,63208)	234 (201.3,266.8)	-5.7 (-11.2,0.1)
Cyprus	3922 (3338,4773)	660.4 (562,797.9)	6772 (5876,7764)	377.8 (329.3,432.4)	-42.8 (-52.5,-33.1)
Denmark	13497 (11412,15540)	177.6 (150.6,205.1)	31945 (28185,35763)	266.7 (235,300.9)	50.2 (38,65)
Finland	8482 (7019,9973)	126 (103.9,148)	18425 (15613,21274)	145.1 (120.5,168.5)	15.2 (7.2,22.9)
France	146507 (130176,162496	180.8 (160.5,199.7)	266719 (232254,297859	174 (151.5,196.1)	-3.7 (-9.2,2)
Germany	300086 (265594,331706	249.2 (219.7,276.9)	635444 (555490,709238	302.9 (266,338.8)	21.5 (12.6,31.2)
Greece	59717 (54719,64585)	424.9 (388.7,459.4)	112985 (100431,122488	435.5 (396.1,475.3)	2.5 (-2.4,7.8)
Iceland	348 (298,399)	120.5 (102.8,138.3)	884 (755,1011)	149.8 (127.2,172.4)	24.3 (14.6,35)
Ireland	9959 (8464,11701)	256.4 (219,299.9)	18990 (16118,22176)	246.7 (207,289.4)	-3.8 (-11.2,4)
Israel	22516 (20420,24257)	480.7 (435.3,518.1)	52713 (46207,58485)	417.3 (366.3,463.5)	-13.2 (-18.5,-8.2)

Italy	198890 (174312,221494	245.8 (215.5,275.2)	328820 (282849,371176	217 (185,247.9)	-11.7 (-17,-5)
Luxembourg	1331 (1185,1491)	264.3 (234.3,295.6)	3013 (2613,3410)	276.4 (240,314)	4.6 (-4.4,15.9)
Malta	1203 (1076,1338)	303 (271.3,336)	2837 (2467,3202)	305.7 (267.1,347.2)	0.9 (-7.2,9.4)
Monaco	121 (100,145)	182.2 (150.3,216.1)	228 (194,266)	233.4 (198.8,273.1)	28.1 (8.3,52)
Netherlands	37878 (32720,42847)	202.1 (174.6,228.7)	82167 (72158,93046)	238.5 (208.3,270.3)	18 (10.4,26.3)
Norway	9013 (7541,10541)	137.2 (113.6,160.3)	17052 (14870,19536)	168.6 (144,194.1)	22.9 (17,30.3)
Portugal	40610 (36847,44376)	330.5 (300.6,360.9)	76569 (67526,85773)	296.5 (262.7,332.8)	-10.3 (-17.5,-2.7)
San Marino	56 (47,66)	167.1 (138.9,195.7)	113 (89,140)	145.8 (113.8,182.3)	-12.7 (-26.7,1.8)
Spain	156194 (141120,171158	309.6 (280.3,339)	243084 (212322,273844	225.4 (196.3,255.5)	-27.2 (-32.5,-21.9)
Sweden	19881 (16791,23071)	141.2 (119.7,164.3)	44455 (38202,50852)	196.3 (169.2,224.9)	39 (26.9,52.4)
Switzerland	21014 (18209,23719)	208.2 (180.6,235.2)	44996 (38424,51285)	228.6 (194.4,262.7)	9.8 (1.7,20.1)
United Kingdom	155905 (129827,180904	192.4 (160.6,223)	258501 (216482,299277	213.4 (177.7,247.3)	10.9 (6.6,16.1)
Southern Latin America	281092 (266405,294525	612.7 (580.9,641.7)	441138 (411345,467913	515.2 (481.8,546.8)	-15.9 (-20.3,-11.5)
Argentina	223942 (212662,235443	705.3 (669.3,740.3)	315536 (295283,335059	573.2 (537.7,608.5)	-18.7 (-23.6,-13.7)
Chile	43420 (40600,46183)	413.7 (384.8,440.4)	104137 (95786,111766)	415.1 (383.4,445.7)	0.4 (-5.9,5.8)
Uruguay	13717 (12910,14498)	364.8 (343.3,385.1)	21441 (19615,23134)	387.1 (357.2,417.5)	6.1 (-0.9,14.3)
Eastern Europe	521705 (463250,581791	207.4 (184.7,230.9)	642467 (562943,726952	204.7 (180.4,232.1)	-1.3 (-7.9,7.1)
Belarus	13038 (10184,15922)	109.6 (86.1,132.8)	21603 (17974,26090)	149.8 (125.8,179.8)	36.7 (22.2,53.3)
Estonia	5689 (5107,6257)	315.4 (284.6,344.8)	11443 (9973,13008)	455.8 (396.1,518.5)	44.5 (28.4,63.9)
Latvia	5859 (5024,6735)	184.2 (159.9,210.5)	9429 (7832,11172)	271.3 (225.8,322.4)	47.2 (28.9,72.3)
Lithuania	7168 (6069,8288)	172.3 (147.2,198)	11654 (9803,13772)	237.6 (201.7,283.9)	37.9 (20.8,61.1)
Republic of Moldova	7738 (6388,9225)	184.4 (151.2,219.8)	12608 (10418,14983)	233.8 (193.3,279.2)	26.7 (15.8,40.8)
Russian Federation	428507 (391751,466416	259.2 (236.9,282)	464004 (409800,529294	213.6 (189.1,243.6)	-17.6 (-23.5,-9.3)
Ukraine	53706 (38096,69878)	84.8 (60.7,108.7)	111725 (89366,135575)	171.8 (138.9,207.4)	102.6 (68.3,150.4)
Central Europe	469644 (436516,504841	342.8 (318.7,368.5)	539982 (472193,612672	266.9 (233.4,306.2)	-22.1 (-28,-14.7)
Albania	9416 (8198,10999)	378.2 (328.6,441.4)	11183 (9088,13561)	303.9 (249.2,366.3)	-19.6 (-34.9,-1)
Bosnia and Herzegovina	13424 (11787,15111)	326.9 (287.9,366.2)	17500 (14014,21236)	311.2 (253.2,378.5)	-4.8 (-23.9,18.3)

Bulgaria	27816 (24930,30650)	274.1 (246.8,301.4)	59631 (50184,71490)	487.9 (408.3,590.7)	78 (47.9,114.4)
Croatia	15608 (14199,16944)	284.1 (259,307)	26558 (22898,31103)	308.8 (265.4,364.9)	8.7 (-5.1,28.4)
Czechia	35113 (31942,38431)	277 (253,303.2)	39158 (33376,45630)	197.7 (167.8,233.1)	-28.6 (-37.2,-18.1)
Hungary	30171 (27070,33010)	235.3 (211.2,256.8)	46215 (39960,53280)	249.3 (214.4,289)	6 (-5.6,19.4)
Montenegro	2342 (1990,2734)	382.9 (325.9,447.3)	3844 (3139,4690)	440.5 (361.7,537.9)	15.1 (-9.5,46.1)
North Macedonia	6763 (5934,7869)	365.5 (320.3,423)	10889 (8783,13799)	373.5 (303.8,461.8)	2.2 (-16.1,24.5)
Poland	166060 (154655,176751	405.7 (378.1,432.2)	136197 (117984,158787	206.2 (177.9,241.2)	-49.2 (-53.7,-43)
Romania	87976 (81527,94928)	348.6 (324,375.7)	92610 (78865,107339)	278 (236.9,323.9)	-20.3 (-30.3,-8.8)
Serbia	42655 (36005,50866)	434.2 (367.7,519.7)	58087 (48712,67910)	374.3 (316.5,434.5)	-13.8 (-31.9,6.5)
Slovakia	19699 (17271,22267)	344.2 (302.5,388.6)	22738 (19458,26211)	265.3 (228.2,305.9)	-22.9 (-34.2,-10.2)
Slovenia	5091 (4580,5635)	220.2 (197.7,243.3)	7512 (6152,9249)	175.2 (142.3,217.9)	-20.4 (-31,-6.5)
Central Asia	176292 (152244,202201	326.1 (278.5,378.8)	427633 (377524,490696	493.2 (436,565.5)	51.3 (37.2,68)
Armenia	4757 (3535,5893)	168.2 (126.2,208.6)	15239 (12675,18395)	380.2 (316.2,457.4)	126 (87.7,189.3)
Azerbaijan	22175 (18576,26606)	374.1 (313.5,447.8)	47430 (37765,57959)	451.9 (364.8,546.7)	20.8 (-1.3,51)
Georgia	15560 (12706,18459)	264.2 (216.6,310.6)	24983 (20472,30628)	478.5 (394.3,587.7)	81.1 (50,121.2)
Kazakhstan	49636 (42866,56925)	351.5 (300,403.8)	80323 (68213,94683)	448.3 (381.6,524.2)	27.6 (10.2,46.5)
Kyrgyzstan	12369 (10585,14240)	346.9 (294.6,401.8)	25313 (21875,29466)	448.3 (386.9,523.7)	29.2 (11.4,50.9)
Mongolia	8119 (6668,9950)	603.2 (498.7,719.3)	13518 (11300,16158)	508.1 (425.4,602.4)	-15.8 (-32.4,1.8)
Tajikistan	6728 (5319,8239)	198.2 (154.9,245.3)	15292 (12007,19273)	217.6 (169.3,274.6)	9.8 (-3.5,28.7)
Turkmenistan	12416 (11128,13743)	475.9 (423.2,536.5)	33906 (27087,41471)	730.9 (586.8,893.3)	53.6 (24,92.9)
Uzbekistan	44533 (35798,54486)	315.3 (247.4,392.7)	171630 (147004,200979	580.8 (501.4,677.5)	84.2 (51.6,124.4)
Central Latin America	779859 (736752,822191)	767.9 (724.2,811.6)	2993750 (2691663,3371708)	1171.1 (1054.8,1316.3)	52.5 (37.6,68.7)
Colombia	122275 (114363,131312	573.8 (533.1,613.7)	233106 (198873,269966	424.4 (362.5,491.3)	-26 (-35.5,-15.5)
Costa Rica	9157 (8313,10118)	468.5 (425.5,515.9)	41809 (37382,46132)	764.5 (683.8,843)	63.2 (46.5,82.9)
El Salvador	28536 (25800,38102)	784.3 (704.6,1063.6)	118020 (88527,144949)	1904 (1419.6,2342.5)	142.8 (50,214.4)
Guatemala	44304 (42312,46649)	920.2 (874.3,970.6)	167359 (143276,194780	1387.5 (1182.2,1617.2)	50.8 (26.4,75.7)
Honduras	11630 (10042,13654)	444.7 (379.4,536.2)	46141 (35816,59163)	682.7 (536.6,871)	53.5 (11.7,99.6)

Mexico	474218 (450937,498550)	932.2 (884.9,983.3)	1940953 (1680240,2260842)	1489.7 (1295.2,1728)	59.8 (40.5,82.9)
Nicaragua	20446 (18371,26070)	922.3 (832.9,1189.3)	86099 (68224,102148)	1596.8 (1282.9,1885.7)	73.1 (21.4,111.9)
Panama	7457 (6800,8224)	442 (401.2,486.7)	36062 (29067,42682)	814.7 (656,963.9)	84.3 (51.1,118.6)
Venezuela （Bolivarian Republic of)	61836 (57241,66347)	527.6 (484.6,568)	324201 (249903,407035)	1087 (843.3,1362.7)	106 (59.7,161.4)
Andean Latin America	190986 (174177,211377	754 (688,838.7)	524246 (434446,625013	872.4 (723.5,1037.9)	15.7 (-4.2,41.2)
Bolivia (Plurinational State of)	45375 (38779,56751)	1123.7 (953.4,1408.5)	121335 (92262,157620)	1307.4 (1008.7,1688.7)	16.4 (-13,59.1)
Ecuador	40275 (37767,42623)	619.3 (581.4,655.6)	155833 (114778,216359	950.4 (705.5,1310.6)	53.5 (14.7,108.7)
Peru	105337 (92303,120681)	713.7 (619.9,822.7)	247079 (184433,311887	720.9 (537.4,910.6)	1 (-25.5,30.4)
Caribbean	161590 (149056,180712	565.1 (523.6,636.6)	385286 (331558,454109	735.8 (631,867.9)	30.2 (12.9,49.6)
Antigua and Barbuda	436 (405,466)	807.8 (751.9,862.9)	1162 (1070,1242)	1116.5 (1028.1,1190.1)	38.2 (25,52.6)
Barbados	1555 (1455,1646)	564.8 (528.5,598.1)	3422 (2743,4181)	732.6 (589.2,903)	29.7 (4.1,61.2)
Belize	848 (796,903)	718.8 (674.5,763.4)	4198 (3725,4677)	1236.1 (1098.8,1377.9)	72 (50.6,93.2)
Bermuda	264 (244,284)	421.4 (389.5,452.8)	482 (414,570)	395.4 (339.7,465.5)	-6.2 (-19.2,10)
Bahamas	1314 (1209,1413)	710.7 (653.6,766.6)	4315 (3520,5314)	1035.6 (849,1270.9)	45.7 (17.8,83.4)
Cuba	29762 (27495,32222)	285.6 (263.5,308.7)	79000 (69453,88900)	436.6 (383.8,494.2)	52.9 (33.8,72.1)
Dominica	502 (447,555)	836.1 (744.6,925.4)	1029 (819,1263)	1295.5 (1035.6,1585.1)	55 (24.1,90.6)
Dominican Republic	25415 (22411,29374)	515.4 (449.4,606.1)	73332 (54273,90211)	701.5 (518.5,864.8)	36.1 (-3.5,74.4)
Grenada	742 (678,812)	1044.7 (958.2,1144)	1684 (1467,1904)	1487.2 (1300.8,1673.6)	42.4 (22.4,63)
Guyana	4232 (3811,4631)	883.2 (800.5,971.4)	10889 (8428,13860)	1574.8 (1227.3,1986.4)	78.3 (34.2,126.8)
Haiti	40217 (31355,58452)	909.5 (697.6,1415.1)	82512 (50633,163742)	916.4 (561.1,1807.6)	0.8 (-33.2,39.2)
Jamaica	11644 (10948,12362)	617.4 (578.5,656.5)	25773 (19982,32671)	831.3 (642.4,1055.4)	34.6 (4.7,73.8)
Puerto Rico	28212 (26770,29738)	793 (753.4,835.2)	51201 (43010,59665)	821.9 (692.5,956.4)	3.6 (-12.6,20.8)
Saint Kitts and Nevis	420 (392,454)	1164.4 (1090.2,1254.5)	914 (747,1082)	1350.4 (1133.2,1575.8)	16 (-5.1,35.5)
Saint Lucia	885 (837,936)	955 (904,1009.7)	2376 (1996,2807)	1041.6 (875.8,1230)	9.1 (-9.4,29.4)
Saint Vincent and the Grenadines	569 (534,610)	737.2 (689.7,789)	1457 (1294,1663)	1083.5 (962.5,1234.4)	47 (28.6,68.4)
Suriname	2433 (2055,2721)	831.3 (717.6,937.4)	7429 (5840,9298)	1181.2 (931.8,1473)	42.1 (7.8,86)

Trinidad and Tobago	6161 (5850,6493)	681.8 (647.7,716.4)	20236 (15316,25658)	1101.3 (836.6,1394.7)	61.5 (22.8,109.2)
United States Virgin Islands	510 (432,606)	563.5 (478.3,667.3)	835 (611,1068)	599.2 (452.3,758)	6.3 (-24.9,42.4)
Tropical Latin America	594892 (560161,631009)	562.7 (527.9,598.7)	1311915 (1220675,1401164)	517 (480.6,552.1)	-8.1 (-12,-4.7)
Brazil	582735 (547944,618485)	565.2 (529.7,600.8)	1273112 (1182007,1361964)	513.8 (477,550.1)	-9.1 (-12.8,-5.9)
Paraguay	12158 (10659,13822)	465.6 (403.4,536)	38803 (31124,48290)	643.4 (516,802.8)	38.2 (4.7,79.1)
East Asia	4372608 (3843678,4996867)	461.3 (406.8,529.1)	6486167 (5538060,7597380)	322.4 (275.4,377.3)	-30.1 (-41.9,-18.5)
China	4195631 (3673738,4813077)	457.7 (402.1,526.9)	6127923 (5184384,7208227)	315.3 (266.6,371.5)	-31.1 (-43.2,-18.7)
Democratic People's Republic of Korea	83478 (63914,105819)	483.4 (373.1,605.9)	153167 (124247,191899	479.2 (390.6,602.6)	-0.9 (-25.2,30.8)
Taiwan (Province of China)	93499 (86835,100493)	604.5 (560.6,649.3)	205078 (181400,224760	502.7 (447,550.5)	-16.8 (-22.7,-11.2)
Southeast Asia	2408834 (2133712,2690991)	751.9 (672.3,850.8)	5703263 (5028657,6329025)	846.3 (753.1,940.5)	12.5 (-2.1,26.6)
Cambodia	48373 (39570,58172)	757.4 (633.3,895.9)	101003 (77788,132516)	733.6 (574.4,943.9)	-3.1 (-30.8,31.3)
Indonesia	875942 (750362,1005150)	664 (576,770.3)	1990496 (1655069,2360240)	760.4 (644.6,906.7)	14.5 (-5.7,36.1)
Lao People's Democratic Republic	36532 (27933,46903)	1380.5 (1067.1,1764.2)	63295 (45478,85994)	1174.6 (854.1,1578.5)	-14.9 (-38,14.9)
Malaysia	77325 (68494,86547)	713.4 (633.7,796.6)	238095 (207099,268811	817.7 (710.6,922.9)	14.6 (-5.1,30.5)
Maldives	1558 (1280,1852)	1347.6 (1152.4,1701.1)	2804 (2338,3335)	719.8 (606.7,852.7)	-46.6 (-58.7,-34)
Mauritius	10096 (9532,10683)	1253.6 (1184.9,1323.6)	38582 (35823,40737)	2196.1 (2043.1,2318.9)	75.2 (61.9,85.9)
Myanmar	292701 (227943,364529	964.3 (759.6,1204.7)	410628 (337703,500939	799.6 (662.6,966.4)	-17.1 (-37.1,11.7)
Philippines	354857 (323905,395725)	919.3 (837.5,1037.4)	1136959 (958347,1307840)	1232.7 (1049.6,1414.5)	34.1 (10.4,56)
Sri Lanka	91811 (79288,106066)	769 (660,894.4)	161739 (117194,209551	628.1 (460.8,813.2)	-18.3 (-42.4,10.8)
Seychelles	522 (466,589)	886.7 (790.4,1000.8)	1424 (1193,1643)	1226.4 (1031.4,1409.8)	38.3 (11.7,67.3)
Thailand	306169 (262369,368081)	746.2 (641,897.8)	865932 (699035,1045232)	859.1 (698.5,1033.1)	15.1 (-13.7,46)
Timor-Leste	3510 (2766,4402)	805.7 (634,1037.6)	8029 (6032,10955)	852 (645.3,1147.7)	5.7 (-23.7,44.4)
Viet Nam	305955 (223778,380082	671.5 (506.4,832.4)	676321 (501845,849831	685.9 (514.2,847.4)	2.1 (-24.4,32.9)

Oceania	22680 (16686,29125)	583.7 (445.8,742)	65815 (55558,77796)	699 (597.9,821.3)	19.8 (-9.5,60.4)
American Samoa	260 (201,353)	953.2 (740.8,1294.2)	938 (740,1163)	1950.9 (1545.9,2408.4)	104.7 (32.9,208.9)
Cook Islands	71 (59,89)	535	151 (120,180)	631.4	18 (-17,58.8)
Micronesia(Federated States of)	595 (425,860)	1017.5 (724.1,1495.1)	1337 (979,1830)	1629.5 (1205.4,2229.3)	60.1 (0.9,147.2)
Fiji	3880 (2939,5283)	849.7 (647.6,1147.1)	10463 (7966,13388)	1313 (1004.2,1661.1)	54.5 (-3.9,137.7)
Guam	535 (470,650)	626.6 (552.2,754.4)	1682 (1452,1875)	846.8 (733.4,946.5)	35.2 (8.8,58.9)
Kiribati	460 (380,548)	970 (797.7,1154.4)	1109 (759,1633)	1285.5 (892.4,1861.8)	32.5 (-15.4,101)
Marshall Islands	212 (144,351)	990.7 (669.1,1662.6)	669 (256,1739)	1587.8 (613.1,4196.4)	60.3 (-20.1,159)
Nauru	70 (47,100)	1204.2 (807.1,1774.9)	127 (95,167)	1785 (1311.6,2476.4)	48.2 (1.4,122.6)
Niue	19 (16,25)	880.2 (706,1130.8)	32 (20,47)	1612.3 (1011.2,2371.9)	83.2 (5.4,174.2)
Northern Mariana Islands	259 (200,334)	1022.8 (833.2,1275.7)	773 (644,914)	1475 (1249.1,1715.8)	44.2 (11,86.5)
Palau	97 (72,125)	880.1 (657.4,1119.2)	293 (214,385)	1382.2 (1017.8,1796)	57.1 (4.2,133.1)
Papua New Guinea	11577 (8052,15381)	451.5 (332.1,589)	36547 (30525,44537)	515.1 (430.9,644.5)	14.1 (-19.2,62)
Samoa	868 (651,1147)	891.3 (674.9,1163.8)	1888 (1466,2394)	1214.4 (948,1524.9)	36.2 (-2.3,96.4)
Solomon Islands	1408 (669,2226)	774.7 (410.4,1191.8)	4011 (3105,5040)	870.9 (699.1,1073.3)	12.4 (-25.8,105)
Tokelau	10 (7,16)	752.8 (553,1180.9)	16 (12,22)	1136.2 (878.9,1548.4)	50.9 (4.9,111.2)
Tonga	294 (223,388)	481.5 (368.9,628.6)	535 (402,689)	639.2 (479.5,818.8)	32.7 (0.1,77.2)
Tuvalu	65 (51,93)	888.1 (693.7,1286.5)	127 (95,175)	1178.5 (885,1617.2)	32.7 (-6.3,81.9)
Vanuatu	551 (376,866)	696.8 (485.1,1113.2)	2161 (1586,3205)	1042 (776.4,1542.2)	49.5 (7,111.1)
North Africa and Middle East	1493457 (1272351,1963220)	759.9 (642.7,1059.2)	3925988 (3427227,4413622)	846.6 (747.2,948)	11.4 (-23.9,35.5)
Afghanistan	94773 (70923,137292)	1274.3 (956,1918.6)	173983 (108001,286182	1343.2 (830.9,2358)	5.4 (-36,59.1)
Algeria	79125 (60711,119848)	593.9 (442.4,962.7)	241373 (197533,295747	724.2 (595.9,868.8)	21.9 (-26.4,70.7)
Bahrain	1611 (1272,2077)	856.9 (670.3,1110.3)	7698 (6382,9087)	963.5 (793.3,1153.7)	12.4 (-25.3,63.1)
Egypt	344984 (287412,482687)	1140.6 (932.6,1686.7)	933318 (767956,1131670)	1501.7 (1248.8,1804.3)	31.7 (-17.2,73.6)
Iran (Islamic Republic of)	139435 (116402,174468	463.6 (372.3,595.6)	356124 (310077,393518	467.5 (406.8,515.1)	0.8 (-27.7,21)
Iraq	94335 (75090,120601)	950.3 (757.7,1205.4)	232396 (157615,296780	940.8 (660.6,1195)	-1 (-35.9,37.9)
Jordan	12490 (10264,15613)	749.9 (617.2,949.5)	54300 (44489,67799)	710.1 (581.4,884.9)	-5.3 (-33.3,24.9)

Kuwait	5748 (5276,6312)	716.8 (647.3,792.7)	11585 (9417,13665)	360.7 (286.1,427.9)	-49.7 (-58.7,-39.8)
Lebanon	16012 (12337,21059)	730.5 (569,955)	39230 (33152,46228)	630.3 (532.3,739.4)	-13.7 (-35.7,16.5)
Libya	15078 (11908,19609)	675.2 (524.4,903.5)	58512 (37581,76498)	1067 (712.7,1379.3)	58 (-14.1,142.9)
Morocco	94039 (70697,146372)	572.7 (420.8,963.5)	289479 (223424,366427	861.5 (664.2,1088.9)	50.4 (-7.3,107)
Palestine	9946 (7801,12853)	979.4 (756.4,1283.1)	23468 (20331,27681)	885.7 (758.8,1049)	-9.6 (-34.1,21.8)
Oman	4965 (3836,6906)	578.2 (442.1,815.4)	19110 (15607,23751)	879 (718.9,1088.5)	52 (4.5,109)
Qatar	1101 (745,1589)	821.9 (512.1,1248.9)	7653 (6095,9741)	757 (614.5,939.6)	-7.9 (-39.6,52.4)
Saudi Arabia	80180 (61535,108291)	1089.8 (832.6,1468.2)	416093 (307761,529386	1762.8 (1351.1,2169.6)	61.8 (-6.6,127.4)
Sudan	76183 (58482,110063)	624.9 (468.4,1022.6)	180786 (136192,237944	770.7 (590,1012.6)	23.3 (-24.6,83)
Syrian Arab Republic	68209 (55197,84454)	968.5 (772.3,1242.8)	126862 (93695,168253)	1020.6 (760,1328.9)	5.4 (-31.5,50.9)
Tunisia	28625 (23139,40343)	534.6 (427.2,765.1)	85581 (62643,113057)	677.1 (500.9,889.4)	26.7 (-24.8,83)
Turkey	288241 (239747,364731	749.2 (624.5,967.7)	552195 (453860,666416	619.4 (509.5,750)	-17.3 (-41.3,3.8)
United Arab Emirates	3892 (2982,5153)	586.6 (434.3,801.6)	29680 (21617,37421)	782.9 (522.7,997.8)	33.5 (-14.7,75.7)
Yemen	33670 (24098,53572)	521.1 (367.1,927.4)	82899 (60825,131865)	510 (372.6,812.5)	-2.1 (-30,36.2)
South Asia	3674401 (3157460,4115106)	509.8 (449.6,573.3)	8443339 (7372315,9681828)	540.6 (473.8,620.4)	6 (-10.1,23.7)
Bangladesh	551460 (306452,697970	604.3 (447.8,702)	577364 (481335,715354	408 (343.9,506)	-32.5 (-44.8,-7.4)
Bhutan	1915 (1398,2531)	571.2 (433,749)	3865 (2821,5112)	600.1 (438.8,786.1)	5.1 (-20.8,40.8)
India	2650925 (2335452,3030444)	481.6 (422.5,547.6)	6487629 (5592187,7501487)	521.8 (451.8,601.8)	8.4 (-12,25.8)
Nepal	65171 (52099,79837)	548.6 (437.4,676.3)	160662 (126887,204193	652.7 (513.5,826)	19 (-10.6,50.1)
Pakistan	404931 (332986,499986)	590.5 (491.7,724.2)	1213820 (972621,1508801)	795.1 (645.5,990.2)	34.6 (5.2,67.2)
Southern Sub-Saharan Africa	202978 (179955,234849	623.9 (548.6,742.1)	551466 (494226,622064	896 (807.3,997)	43.6 (19.2,61.7)
Botswana	4159 (3143,5808)	645.6 (493,883.8)	11766 (9282,16126)	734.5 (596.1,978.2)	13.8 (-15.4,51.2)
Lesotho	5121 (4128,6439)	560.6 (450.9,707.1)	14547 (10549,19456)	1221.5 (901,1605.1)	117.9 (58.2,186.5)
Namibia	4420 (3529,5944)	613.6 (493.2,821.7)	10401 (7883,13859)	695.8 (539.9,902.1)	13.4 (-13.5,46)
South Africa	155415 (136560,179958	616.7 (540.4,733.3)	415624 (377739,462537	862.3 (784.9,953.7)	39.8 (19.3,55.8)
Eswatini	3616 (2910,4479)	1010.5 (813.5,1237.9)	9663 (6172,13306)	1431 (945,1925)	41.6 (-0.4,94.7)

Zimbabwe	30248 (24565,39272)	664 (538,847)	89465 (68552,116945)	1084.3 (848.3,1401.9)	63.3 (18.5,112)
Western Sub-Saharan Africa	1112629 (952298,1275995)	928.7 (800.3,1067.1)	2437269 (1992652,2893649)	930.7 (788.4,1081.1)	0.2 (-13.2,13.9)
Benin	29653 (25048,34516)	999.3 (868.4,1155.7)	71721 (57427,87409)	990.1 (808.6,1210.3)	-0.9 (-20,22)
Burkina Faso	60272 (50415,71118)	1014.2 (852.5,1190.6)	140312 (112697,173625	1094.7 (897.5,1322.3)	7.9 (-15.9,35)
Cameroon	80907 (63460,102530)	1348.1 (1083,1721.9)	224422 (163790,306794	1320 (973,1795.1)	-2.1 (-22.9,28)
Cabo Verde	1204	462.9	3178	677.4	46.3 (5,85.6)
Chad	28291 (22969,36811)	736.8 (598.1,968.7)	73527 (56687,98420)	840.5 (646.7,1175.4)	14.1 (-9.7,44)
CÃ´te d'Ivoire	62444 (50103,77072)	1012.2 (827.3,1226.8)	153321 (116483,194558	1019.7 (809.3,1277.2)	0.7 (-18.7,29.2)
Gambia	4626 (3620,5727)	892.6 (714,1097.6)	13852 (10535,17707)	1074.8 (822.4,1368.1)	20.4 (-11.9,62)
Ghana	67702 (52675,92629)	772.8 (608.9,1076.7)	237458 (184981,297856	1145 (904.6,1420.1)	48.2 (-1.5,98.7)
Guinea	39480 (32723,48547)	900.2 (741.3,1118.7)	67059 (52117,90058)	897 (697.8,1221.3)	-0.4 (-23.3,28.6)
Guinea-Bissau	8272 (6561,10146)	1414.4 (1129.6,1709.7)	12715 (9952,16050)	1220.6 (970.4,1525.5)	-13.7 (-33.3,14.3)
Liberia	20964 (16740,25988)	1241 (1006.8,1562.2)	35243 (26717,46785)	1197 (916.2,1548.1)	-3.5 (-29.9,30.8)
Mali	56774 (47423,68232)	1040.3 (873.6,1283.9)	114837 (94129,145340)	950.5 (780.4,1210.6)	-8.6 (-26,14.4)
Mauritania	13123 (10398,16336)	1080.7 (859.4,1353.4)	23187 (16553,31976)	937.8 (665.7,1292.5)	-13.2 (-32.7,12.4)
Niger	41923 (33643,50607)	842.5 (698.1,1076.1)	80122 (59677,120176)	687.8 (523.4,1007.5)	-18.4 (-37.6,7)
Nigeria	506251 (409099,599064)	881 (719.4,1053.3)	1007285 (771422,1268598)	824.4 (662.5,1015.2)	-6.4 (-24.9,14.4)
Sao Tome and Principe	1102 (882,1290)	1349 (1113.6,1530.2)	2097 (1609,2634)	1636.6 (1240,1979.7)	21.3 (-9.9,54.3)
Senegal	49306 (41083,60783)	1065.1 (898.2,1309.7)	95390 (73587,131487)	1027.6 (799,1391.7)	-3.5 (-26.3,30.5)
Sierra Leone	24287 (18928,30087)	832.1 (664.2,1021.9)	39388 (29974,52547)	770.1 (604.3,1010.8)	-7.5 (-28,19.1)
Togo	16010 (13190,19324)	839.2 (706.9,1015.6)	42127 (31777,54924)	888.2 (691.8,1154.8)	5.8 (-17.7,36.6)
Eastern Sub-Saharan Africa	1117669 (975468,1248904)	1095.2 (965.9,1255)	2047629 (1792019,2374164)	948.4 (838.7,1090.4)	-13.4 (-23,-0.9)
Burundi	31943 (25440,39942)	1032.6 (815.3,1304.7)	52752 (39451,75471)	837.3 (623.9,1184.7)	-18.9 (-39.5,10.3)
Comoros	2494 (1867,3109)	984.8 (763.6,1217)	5242 (3955,6654)	990.7 (748.1,1260.4)	0.6 (-26.1,38.2)
Djibouti	1395 (1048,1830)	727.5 (561.1,939.8)	6952 (5050,9547)	959.1 (723.5,1275.5)	31.8 (-9.9,84.5)
Eritrea	15112 (11352,20868)	900.2 (680.9,1231.1)	32532 (22413,51729)	930.2 (649.1,1463.5)	3.3 (-18.9,35)

Ethiopia	466721 (355203,540047	1765.7 (1400.4,2039.4)	524752 (440481,620029	966 (806.9,1140.3)	-45.3 (-55.5,-31.9)
Kenya	66436 (54525,94453)	608.9 (488.9,921.4)	230896 (188910,304540	863.7 (701.7,1139.4)	41.8 (12,79.5)
Madagascar	47382 (38780,60549)	702.8 (568.5,945.4)	100664 (76256,129965)	678.7 (508.2,884.3)	-3.4 (-28.2,25.1)
Malawi	56194 (46192,66313)	1003.2 (830,1214.1)	110135 (88010,133712)	1114.9 (915.1,1338)	11.1 (-13.4,38.5)
Mozambique	59057 (48400,73912)	681.4 (559,901.3)	141035 (107847,186077	882.2 (684.6,1181.5)	29.5 (-6.2,73.3)
Rwanda	48445 (39539,56316)	1255.6 (1043.6,1472.4)	65862 (48112,87152)	891.8 (663.5,1176.6)	-29 (-46.8,-5.2)
Somalia	42592 (31540,57931)	1182.7 (867,1615.4)	109717 (77473,161379)	1218.6 (853.7,1741)	3 (-22,37.1)
South Sudan	33407 (25693,43506)	1014.3 (768.8,1354.6)	69366 (50852,90453)	1345 (1002.1,1742.3)	32.6 (-11.3,83.8)
United Republic of Tanzania	131082 (109800,160936	903.1 (755.1,1140.9)	288849 (234235,364048	899.3 (731.7,1124.4)	-0.4 (-24.7,32.3)
Uganda	65235 (50387,82208)	764.5 (582,980.3)	183294 (144104,239789	919.2 (725.5,1193.4)	20.2 (-12.3,64.7)
Zambia	49375 (39792,57931)	1179.3 (995.9,1379.6)	123798 (84564,188445)	1289.7 (932.6,1817.4)	9.4 (-22.8,69.5)
Central Sub-Saharan Africa	349929 (293393,410516	1158.7 (983.4,1367)	783700 (622595,996532	1124.7 (899,1436.1)	-2.9 (-23.8,24.9)
Angola	60560 (47304,74043)	1068.6 (834.9,1327)	158051 (120480,207619	1011.8 (761.7,1315)	-5.3 (-29.3,26.7)
Central African Republic	20433 (16924,24299)	1327.3 (1106,1592.6)	39107 (28354,53349)	1303.3 (972.4,1758.2)	-1.8 (-24,27.7)
Congo	19457 (15403,23362)	1483.5 (1157.4,1769.5)	44198 (31384,57320)	1365.6 (964.3,1712.3)	-7.9 (-27.7,15.6)
Democratic Republic of the Congo	238276 (194665,290653	1138.9 (935.7,1393.9)	513717 (387253,677593	1112.3 (844.2,1452.8)	-2.3 (-28.4,35.2)
Equatorial Guinea	3014 (2404,3713)	1226.6 (976.6,1504.5)	9444 (6004,13862)	1323.1 (834.3,1860.8)	7.9 (-31.5,57.5)
Gabon	8190 (6669,9730)	1304.8 (1072.4,1558)	19184 (11205,25666)	1670.8 (951.9,2188.5)	28.1 (-15.3,68.2)
